# The structure of a β_2_-microglobulin fibril suggests a molecular basis for its amyloid polymorphism

**DOI:** 10.1038/s41467-018-06761-6

**Published:** 2018-10-30

**Authors:** Matthew G. Iadanza, Robert Silvers, Joshua Boardman, Hugh I. Smith, Theodoros K. Karamanos, Galia T. Debelouchina, Yongchao Su, Robert G. Griffin, Neil A. Ranson, Sheena E. Radford

**Affiliations:** 1 Astbury Centre for Structural Molecular Biology, School of Molecular & Cellular Biology, Faculty of Biological Sciences, University of Leeds, Leeds, LS2 9JT UK; 2 Department of Chemistry and Francis Bitter Magnet Laboratory, Massachusetts Institute of Technology, Cambridge, MA 02139 USA; 3 Present Address: Department of Chemistry & Biochemistry, Florida State University, 95 Chieftan Way Rm. 118 DLC, Tallahassee, FL 32306-4390 USA; 4 Present Address: Laboratory of Chemical Physics, National Institute of Diabetes and Digestive and Kidney Diseases, NIH, Bethesda, MD 20892-0510 USA; 5 Present Address: Department of Chemistry and Biochemistry, University of California San Diego, La Jolla, CA 92093 USA; 6 Present Address: Merck Research Laboratories, Merck & Co., Inc., Kenilworth, NJ 07033 USA

## Abstract

All amyloid fibrils contain a cross-β fold. How this structure differs in fibrils formed from proteins associated with different diseases remains unclear. Here, we combine cryo-EM and MAS-NMR to determine the structure of an amyloid fibril formed in vitro from β_2_-microglobulin (β_2_m), the culprit protein of dialysis-related amyloidosis. The fibril is composed of two identical protofilaments assembled from subunits that do not share β_2_m’s native tertiary fold, but are formed from similar β-strands. The fibrils share motifs with other amyloid fibrils, but also contain unique features including π-stacking interactions perpendicular to the fibril axis and an intramolecular disulfide that stabilises the subunit fold. We also describe a structural model for a second fibril morphology and show that it is built from the same subunit fold. The results provide insights into the mechanisms of fibril formation and the commonalities and differences within the amyloid fold in different protein sequences.

## Abstract (web summary)

Impaired kidney function can lead to an increase of β_2_-microglobulin (β_2_m) serum levels, which can cause β_2_m aggregation and amyloid fibril formation. Here the authors combine cryo-EM and magic angle spinning NMR measurements to determine the structure of a β_2_m fibril and they also present the low resolution model of a β_2_m fibril with a different morphology.

## Introduction

Amyloid fibrils are fascinating protein assemblies that play a central role in many devastating diseases^1^. They also have functional roles from microbes to man^2,3^ and offer the opportunity to generate novel materials with new properties^4–6^. Despite these varied functions, all amyloid fibrils have a common architecture based on a cross-β structure^7^. Despite the first identification of the cross-β motif more than 50 years ago^8^, the structure of amyloid eluded high-resolution structural definition for all but the smallest of peptide assemblies^9^. This raised the question of how many structures conform to the canonical cross-β fold; how different sequences can assemble into this same fold family; and how the structure of amyloid fibrils generated in vitro relate to their counterparts formed in situ. Recent developments in magic angle spinning (MAS)-NMR and cryo-EM have seen an end to this impasse, with high-resolution structures of fibrils formed from Aβ_42_ and α-synuclein in vitro, and tau fibrils ex vivo being reported in the last year^10–14^. These proteins are all intrinsically disordered in their native, functional states, and hence amyloid assembly involves peptide ordering into the cross-β fold. Of the >50 currently known amyloid precursors, however, almost half are initially folded, including light chains, serum amyloid A, prions, and β_2_-microglobulin (β_2_m)^15^. How the amyloid structure(s) of these proteins relate to the structures of their folded, functional forms and to the architecture of amyloid fibrils assembled from intrinsically disordered precursors remained unclear.

In its native, functional state, β_2_m forms a canonical, seven β-stranded immunoglobulin fold that chaperones the folding and assembly of class 1 major histocompatibility complex (MHC-1). MHC-1 is found on the surface of all nucleated cells and is essential for immunity^16^. Following dissociation from MHC-1, β_2_m is normally cleared by the kidneys, but in patients with impaired kidney function who are undergoing long-term haemodialysis, serum levels of β_2_m rise as much as 40-fold^17^. This leads to aggregation of β_2_m and its deposition as amyloid fibrils in the joints^18^. The associated disease, dialysis-related amyloidosis (DRA), is marked by debilitating arthritis and bone damage^17^. The major protein component of amyloid deposits in DRA is wild-type β_2_m (~70%), together with several truncation products, the most prominent of which involves deletion of six amino acids from the N-terminus of the protein, generating the highly amyloidogenic variant, ΔN6 (~30%)^19^. Fibrils formed from β_2_m in vitro have been shown to disrupt membranes^20,21^, perturb endosomal–lysosomal trafficking^22^, and reduce the viability and/or function of monocytes, chondrocytes, osteoblasts, and osteoclasts^23^, implicating fibril deposition in disease.

The formation of amyloid fibrils from β_2_m in vitro at physiological pH requires partial unfolding, specifically involving the *cis*–*trans* isomerisation of Pro32, and the formation of an unstable, non-native state that nonetheless retains its immunoglobulin fold^24,25^. Retention of the single disulfide bond which links residues Cys25 and Cys80 in the native state is also required for amyloid formation in vitro^26^, and this disulfide is intact in fibrils in vivo^27^. Despite being the major component of amyloid fibrils in DRA deposits, wild-type β_2_m is resistant to aggregation in vitro unless the protein is first unfolded by lowering the pH or adding co-solvents or other additives. Fibrils generated at low pH in vitro bind collagen, glycosaminoglycans, and serum amyloid P component, akin to their biological counterparts^28,29^ and possess similar secondary structure as judged by FTIR^30^. However, despite this plethora of studies, including preliminary analysis using MAS-NMR^31^, the structure of β_2_m amyloid remained unsolved, leaving unanswered questions as to how the initially all anti-parallel β-sheet structure of the native protein is remodelled when forming an amyloid fibril. Here, we created fibrils from β_2_m at low pH in vitro and, by combining assignment and distance constraints obtained using MAS-NMR with cryo-EM analysis, we solved their structure to near-atomic resolution. The results reveal the conformational changes that occur as the protein forms amyloid, and show how different packing of the same subunit fold generates β_2_m fibril polymorphism.

## Results

### β_2_m fibrils contain a single subunit fold

**Fig. 1:**
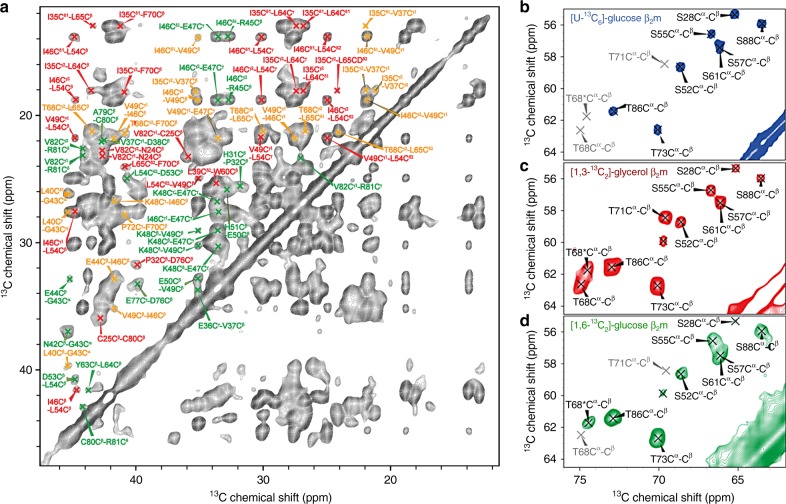
MAS-NMR spectra of β_2_m fibrils show a single subunit structure. **a** Excerpt of a 2D ^13^C–^13^C MAS spectrum of uniformly [^13^C/^15^N]-labelled β_2_m fibrils using 15 ms PAR mixing recorded at a field strength corresponding to *ω*_0H_/2*π* = 900 MHz, *T* = 268 K, and *ω*_r_/2*π* = 20 kHz. *τ*_mix_ = 15 ms, with an 83 kHz ^1^H decoupling field applied during acquisition. **b**, **c**, **d** C^α^–C^β^ correlations of Ser/Thr residues in samples differentially labelled with ^13^C, as indicated for each plot. For each Ser and Thr only one correlation should be present in a monomorphic sample. Since β_2_m has 5 Thr and 9 Ser residues, a total of 14 resonances should be observed. For Ser, 6/9 expected peaks (S28, S52, S55, S57, S61, and S88) are observed. S33 is not resolved, since its C^α^ and C^β^ chemical shifts are identical, i.e. the cross-peak overlaps with the diagonal peak. S11 is unobserved since it is part of the region of intermediate flexibility. We observed a weak peak that is not connected to any other residues and is believed to stem from S20. For threonine, 4 cross-peaks are expected (T68, T71, T73, T86), since T4 is part of the very flexible N-terminus and is not observed. However, 5 cross-peaks are observed. The cross-peak of T68 exhibits doubling, presumably reflecting local structural perturbations for different polymorphs (see main text). Grey peak designations are included as a reference for missing peaks in this spectrum or which lie below the plotting level used. These differences are a result of the different labelling schemes and mixing efficiencies

MAS-NMR and electron paramagnetic resonance (EPR) studies can both provide precise distance constraints to describe the structural  properties of proteins in solution^32,33^. Previous MAS-NMR and EPR studies have shown that the N-terminal ~10 residues of β_2_m in fibrils generated at low pH in vitro are highly flexible^31,34,35^, consistent with limited proteolysis^36^, and H/D exchange data^37^. To gain more insight into the conformation of β_2_m within the core of these amyloid fibrils, we conducted an extensive series of 2D and 3D MAS-NMR studies on fibrils grown from β_2_m monomers labelled specifically or uniformly with ^15^N and ^13^C (Methods). Using these different labelling strategies, we were able to assign >90% of the heavy atoms for residues F22–S88 consistent with these residues forming the ordered core of β_2_m in the fibrillar state (Fig. 1). Crucially, with the exception of T68, a single set of resonances was detected for residues within this core region (Fig. 1b–d and Supplementary Figure 1), unambiguously showing that the fibrils contain β_2_m subunits that have a single well-defined tertiary structure.

### Cryo-EM structure of β_2_m amyloid fibrils

**Fig. 2:**
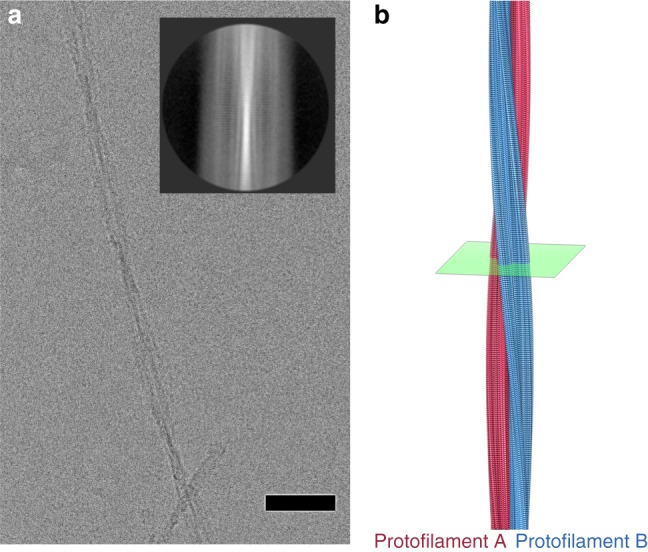
The cryo-EM structure of a two protofilament β_2_m amyloid fibril. **a** Raw cryo-EM image of a β_2_m fibril in vitreous ice. The scale bar is 50 Å in length. Inset: an average of fibril segments shows periodicity which is perpendicular to the fibril long axis with a ~4.8 Å spacing. **b** One turn of the β_2_m amyloid fibril, with the two protofilaments coloured red and blue. See also Supplementary movie 1

β_2_m fibrils generated under identical conditions were also analysed by cryo-EM. The resulting images revealed untangled, long, and straight fibrils (Fig. 2). Despite the unambiguous evidence from MAS-NMR for a single conformation of β_2_m in the fibrillar state, when visualised directly the fibrils display a broad range of morphologies, characterised by different widths and crossover lengths. The most common type observed (56% of the fibrils analysed (Supplementary Table 1)), had easy-to-identify crossovers and appeared to be formed from two protofilaments (Fig. 2a). Computational averaging of >90,000 segments generated classes that displayed the 4.8 Å repeat characteristic of stacked β-strands in amyloid (Fig. 2a (inset)). The fibrils exhibit poor helical order with large variations in pitch. This is exhibited by large variations in crossover distances (the distance between the points where a visible cross-over is observed in the twisting fibril (equal to half the true helical pitch)), of between 100 and 150 nm. This variation is observed both within individual fibrils and between different fibrils in the same preparation, consistent with previous observations^38^. After extensive 2D and 3D classification to produce a dataset of fibril segments with consistent crossover length, followed by 3D refinement with helical symmetry in RELION^39,40^, we generated a cryo-EM structure for a two protofilament fibril at 3.9 Å resolution (Fig. 2b and Methods).

### The β_2_m amyloid fibril has two protofilaments

**Fig. 3:**
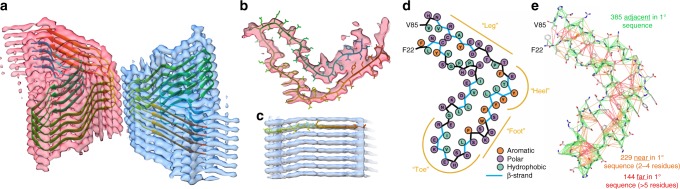
The structure of a β_2_m amyloid fibril. **a** Oblique view of the cryo-EM map, with four layers of a de novo atomic model built into each protofilament (coloured pink and blue). The view matches the green section in Fig. 2b. **b** Cross-section and **c** side view of a single layer of the atomic model, showing the fit of model to density across the β_2_m subunit. **d** Schematic view of a single β_2_m subunit showing polar (purple), hydrophobic (cyan), and aromatic residues (orange) in the fibril core. The position of β-strands is indicated by a blue backbone in the schematic, together with the locations of the toe, foot, heel, and leg terminology used to describe the structure in the main text. **e** Distance constraints from the MAS-NMR data mapped onto the atomic model derived by cryo-EM

**Fig. 4:**
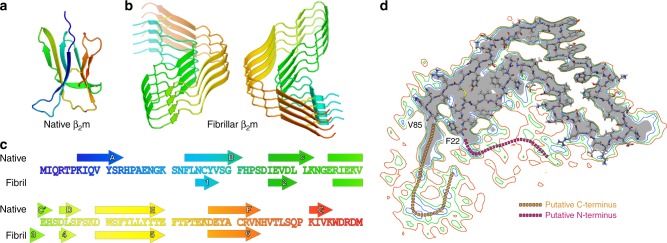
The structure of β_2_m in its native and fibrillar states. The strands of native β_2_m (**a**) are labelled A–G, and those of the fibrillar β_2_m subunit (**b**) are labelled 1–6. Each structure is identically coloured from N-terminus (blue) to C-terminus (red). Residues in the N-terminal (Strand A, blue) and C-terminal (Strand G, red) strands of the native structure are disordered and not present in the core of the fibrillar subunit. **c** β-strands in the fibrillar subunits coincide remarkably well to β-strands B–F in the native fold, although the B and C strands are shorter. **d** The N- and C-terminal regions of the β_2_m fibril are disordered. The atomic model for the ordered portion of the fibril superimposed over slices through the EM density perpendicular to the fibril axis at varying contour levels, showing less-ordered regions that must contain the N- and C-terminal residues. The grey density is at a contour level of 0.04, followed by a contour at 0.035 (blue), 0.030 (green), and 0.025 (red), which show the position of weaker, less-ordered density

The cryo-EM density revealed that the β_2_m fibril is comprised of two protofilaments arranged in parallel (Fig. 3b) and is built from discrete layers of density. Each layer is related by a twist of −0.608° and rise of 4.83 Å, giving the fibril a left-handed twist, which was verified using scanning electron microscopy (SEM) (Supplementary Figure 2). Each layer contains two β_2_m molecules (one per protofilament) resulting in a mass-per-length of 53.3 kDa/nm, consistent with previous scanning (STEM) and tilted beam (TB-TEM) electron microscopy measurements^38,41^. The quality of the EM density was sufficient to build an atomic model for the ordered core of the β_2_m subunit (Fig. 3). The subunits in each protofilament have an identical conformation that is related by a 180° rotation along the fibril long axis (C_2_ symmetry). The subunits within the two protofilaments are therefore stacked in-register with each other. The β_2_m subunits have an ordered, L-shaped core formed by residues 22–85 (Fig. 3d). This conformation is consistent with the MAS-NMR data, including a total of 1157 unique distance constraints (Fig. 3e; Supplementary Figure 3; Supplementary Tables 2–5). These data provide an orthogonal validation of the cryo-EM structure of the amyloid subunits presented here, including the length and position of β-strands (Fig. 4a–c). These constraints include intra-residue contacts (tabulated in Supplementary Table 2), as well as contacts between atoms in adjacent residues in the primary sequence (green in Fig. 3e; Supplementary Table 3), between atoms in residues close in the primary sequence (2–4 residues apart; orange in Fig. 3e; Supplementary Table 4), and between atoms in residues far apart in the primary sequence (>5 residues; red in Fig. 3e; Supplementary Table 5). Overall, these results show that in the fibril, the 100-residue β_2_m molecule contains a dynamic region (M0 to ~V9), a region containing residues with intermediate flexibility (Y10–N21 and Q89–M99) and a rigid core (F22–S88), consistent with previous EPR^35^, N-ethylmalemide-labelling^35^, H/D exchange^19,37^, and proteolysis^19,36^ studies.

### Similar secondary structure elements build distinct tertiary folds

In its native functional state, β_2_m has an immunoglobulin fold that contains seven anti-parallel β-strands (A–G; Fig. 4a, c)^42^. By contrast, each β_2_m molecule in the fibril has six β-strands (strands 1–6; Fig. 4b, c) which are stacked parallel and in-register in the fibril core, consistent with previous MAS-NMR^31^ and EPR^35^ data. Remarkably, these β-strands correspond extremely well in position to β-strands B–F in the native protein, although the length of the fibrillar β-strands 1 and 2 are shorter than the corresponding β-strands B and C in the native β_2_m fold (Fig. 4b, c). Thus, the fibrillar conformation is built from substantially the same secondary structural elements as the native conformation. Interestingly, recent MAS-NMR studies of a naturally-occurring variant of β_2_m (D76N), which causes systemic amyloid disease in the absence of kidney failure^43^, has a different number and location of its β-strands^44^. This suggests that the fibrils formed from D76N monomers, and perhaps those of other variants such as ΔN6^34^, may adopt fibrillar structures different from those presented here for the wild-type protein. This is further supported by the fact that whilst D76N and ∆N6 β_2_m assemble rapidly into fibrils at pH 6–7, low pH was used here to generate fibrils from the wild-type protein since this sequence does not aggregate at neutral pH unless destabilised by the addition of metal ions, detergents, or co-organic solvents^45^. In accord with this view, previous results have shown that β_2_m fibrils formed at low pH rapidly depolymerise by shedding oligomers from their ends when the pH is raised^46^, presumably because the deprotonation of E44 and D76 in the fibril core destabilises the parallel in-register stacking of its β-strands. The N-terminal 22 residues and C-terminal 14 residues are disordered in the cryo-EM structure (Fig. 4d), and β-strands A and G in the native structure which lie in these regions are not seen at high resolution in the cryo-EM structure. However, additional weak density corresponding to these regions is observed, consistent with the residues involved being poorly ordered, but localised close to the fibril surface (Fig. 4d).

### Molecular interactions stabilising the subunit and fibril

**Fig. 5:**
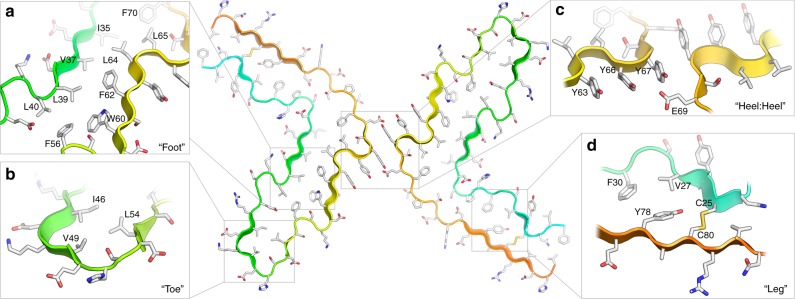
The atomic model of a two protofilament β_2_m fibril. The central panel shows two β_2_m subunits, and represents a cross-section through a layer of the EM reconstruction, perpendicular to the long fibril axis. Each β_2_m subunit is L-shaped, stacked in register, perpendicular to the fibril long (C_2_) axis, and related by a 180° rotation about the C_2_ axis (in the centre). The polypeptide is shown in cartoon representation, coloured from the N-terminus (i.e. M0 would be deep blue) to the C-terminus (M99 would be deep red). Side-chains are coloured in a CPK scheme. **a** A steric zipper in the foot; **b** a β-turn in the toe; **c** disulfide-containing region; and **d** interface between protofilaments showing π-stacking. See also Supplementary movie 2

The MAS-NMR and cryo-EM data collectively allowed us to build and validate a unique structural model for the fibrillar subunit of β_2_m that reveals details of both the intra- and inter-subunit interactions that stabilise the protofilament and fibril structures (Fig. 5). Features found in other amyloid fibrils were observed^10–14^, as well as interactions unique to β_2_m fibrils. The canonical, parallel in-register cross-β structure is maintained down the fibril long axis by networks of hydrogen bonds between backbone atoms in the β-strands, supported by extensive π-stacking interactions between the aromatic residues Phe22, Tyr26, Phe30, Phe56, Trp60, Phe62, Phe70, and Tyr78 along the length of the core (Supplementary Figure 4), as observed in other amyloid fibrils^10–14,47–49^. The β-strand-containing regions (strands 2 and 5) in the foot of the subunit (for nomenclature, see Fig. 3d) are stabilised by a classic steric zipper formed by residues Trp60–Leu39–Phe62–Val37–Leu64–Ile35 (Fig. 5a) in which the surface shape complementarity^50^ is as high as that seen in zippers formed from small peptides (*S*_c_ = 0.80–0.86^9^; Supplementary Figure 5). This zipper is capped on each end by hydrophobic pairings between Phe56/Leu40 and Leu65/Phe70 (Fig. 5a). The adjacent ‘toe’ region of the subunit (residues 46–54) is stabilised by hydrophobic interactions between Ile46, Val49, and Leu54 (Fig. 5b). The ‘leg’ (residues Thr71–Arg68 and Phe22–Ser33, including β-strands 1 and 6) contains the intramolecular disulfide bond (Cys25–Cys80) that is found in the native protein and is required for fibril formation in vitro^51^ and in vivo^52^. This region is further stabilised by hydrophobic interactions between Phe30, Val27, and Tyr78 (Fig. 5c). Residues Glu44 and Asp76 which will be protonated at the pH at which these fibrils were grown, are packed in the core of the fibril, while other charged side chains are solvent exposed. Residue Pro32, which is in a *cis* conformation in the native β_2_m fold, is in a *trans* conformation in the fibril, in accordance with the MAS-NMR data^32^. Finally, each subunit is stabilised by an unusual face-to-face, three-ring π-stack (Tyr63, Tyr66, and Tyr67) perpendicular to the fibril axis which kinks the polypeptide backbone in the region where the protofilaments meet (Fig. 5c). The average centroid–centroid distance between rings of Tyr63/Tyr66 and Tyr66/Tyr67 is ~4.8 Å. This perpendicular π-stacking not only stabilises each subunit, but provides additional stabilising interactions between protofilaments, which associate via the heels of two subunits, forming an interface that is unlike that seen for any other amyloid fibril to date. The main stabilising interaction appears to be an inter-molecular H-bond between Tyr67 and Glu69 (Glu69 is protonated at low pH), facilitated by the kinked conformation of the polypeptide chain (Fig. 5c). The distance between Tyr67 rings across the protofilament interface is ~7.6 Å, suggesting that π-stacking (and perhaps π-amide) interactions across the interface would make only a minor contribution to overall fibril stability (Supplementary Table 6). However, given that this small stabilisation would be multiplied by the enormous number of these interactions running down the fibril axis, its contribution is likely to be significant.

### β_2_m fibrils have a wide range of morphologies

**Fig. 6:**
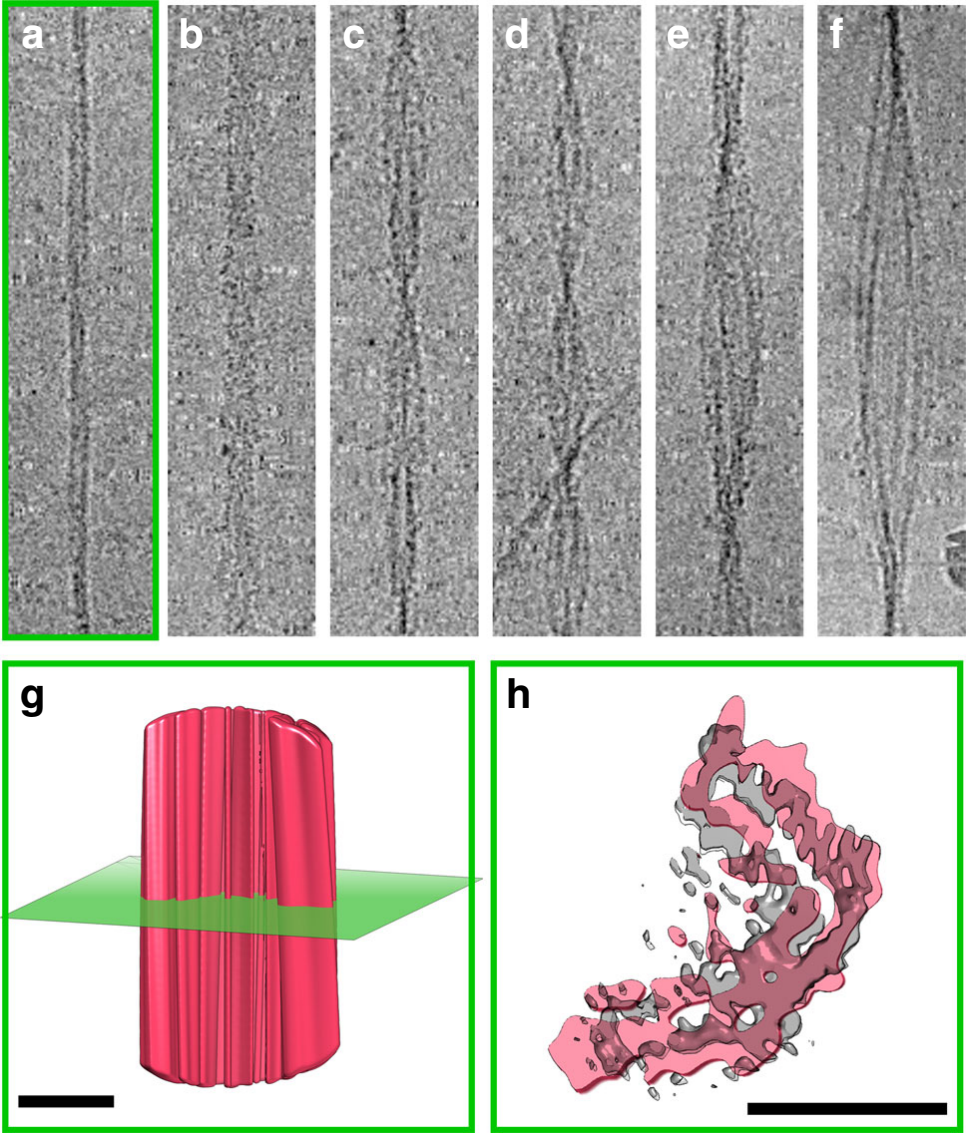
Variety in fibril morphology. Six different morphologies were identified in the non-entangled fibrils constituting the cryo-EM dataset. These range in size from apparent single protofilaments (**a**), through a series of apparent two protofilament fibrils (**b**–**d**), the structure of one of which has been described here at 3.9 Å resolution (panel **c**), to assemblies of multiple protofilaments (**e**, **f**). Each panel (**a**–**f**) is 500 Å wide. Side view (**g**) and cross-section through (**h**) a low-resolution reconstruction of morphology (**a**) showing density consistent with an L-shaped subunit. Each scale bar in panels (**g**) and (**h**) is 50 Å in length

Detailed analysis of the cryo-EM dataset enabled at least six different fibril morphologies with varying diameters and twists to be identified within the same sample (Supplementary Table 1, Fig. 6a–f), despite only a single set of resonances being observed by MAS-NMR (Fig. 1). However, this underestimates the true extent of heterogeneity, since many further examples were observed by cryo-EM in which all fibril types were twisted together or intertwined (Supplementary Figure 6), including large-scale aggregates which are also observed on the EM grids. Such heterogeneity defies classification beyond ‘higher order aggregate’. To explore the molecular basis for the different morphological classes observed, we determined 3D reconstructions (see Methods) for a second fibril morphology, although at much lower resolution owing to greater heterogeneity and the relative scarcity of this fibril type in the dataset. These thin fibrils (Fig. 6a) have the same L-shaped subunit cross-section described above (Fig. 6g, h) and are formed from a single protofilament. The resolution of this structure is difficult to assess, owing to the very high symmetry applied during reconstruction and the lack of features which are resolvable in the fibril structure at intermediate resolution. However, the gap between the two β-strands within a subunit is poorly resolved, suggesting a resolution of ~10 Å. Kinetic studies of fibril growth using AFM showed that these fibrils are able to assemble into thicker fibrils on an hour-to-day timescale (Supplementary Figure 7). The thicker fibrils (Fig. 6d–f) appear to be formed by the intertwining of two fibrils of type C described above (i.e. they create a four protofilament fibril). The rarity of these fibril types precluded structural modelling in more detail. However, given that a single NMR resonance is observed for each residue in the fibril core all polymorphs must be built from the same canonical, L-shaped building block. Notably, the C^α^–C^β^ resonance of Thr68 is the only resonance that is doubled in the NMR spectra (Fig. 1b–d). This residue lies in the kinked region at the heel of the subunit (Fig. 2d) and presumably reports on local perturbations at this site in the different fibril polymorphs.

## Discussion

**Fig. 7:**
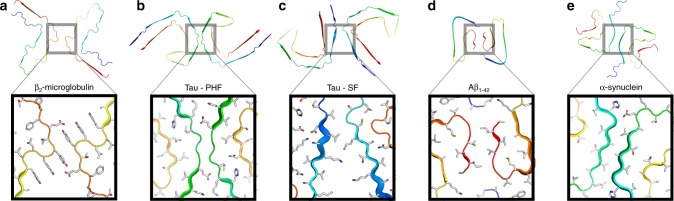
Inter-protofilament interactions in cryo-EM structures of amyloid fibrils. Comparisons of the subunit structures (upper) and interfaces between protofilaments (boxes) of **a** β_2_m, Tau variants^11^ that form **b** paired helical filaments (PHF) and **c** straight filaments (SF), **d** Aβ_1–42_^10^ and **e** α-synuclein^13^. The β-strands present in each structure are coloured blue. Fibrils were analysed from the following PDB files: Tau PHF (5o3l), Tau SF (5o3t), Aβ_1–42_ (5oqv), and α-synuclein (6h6b)

Comparing the β_2_m fibril structure presented here with other recent high-resolution structures of amyloid fibrils (Fig. 7 and Supplementary Table 7)^10,12–14,47–49,53–55^ highlights both commonalities in structure between members of the amyloid fold family, as well as key distinctions that reflect the disparate protein sequences involved. The latter may underlie the different locations of fibril deposition in vivo, and hence the different diseases caused by amyloid deposits formed from the same protein precursor^1,15^. Despite the lack of any sequence similarity, the β_2_m fibrils described here bear a marked resemblance to the paired helical and straight filaments (PHF and SF) of Tau^11^ with their C-shaped subunits (Fig. 7), which contrasts markedly with the straight stacked β-strand architecture seen in fibrils formed from short 5–10 aa peptides^9^. Similar convoluted C-shaped subunits have been seen in fibrils formed from Aβ_42_ and α-synuclein (Fig. 7). However, the ordered core of β_2_m subunits is L-shaped, and stabilised by different interactions than those seen in other fibrils. For example, the toe of the Tau PHF subunit is formed by a β-helix, but in β_2_m fibrils this region involves a turn stabilised by hydrophobic packing of Ile46, Val49, and Leu54 (Fig. 5). β_2_m amyloid fibrils contain a steric zipper motif, in common with all high-resolution amyloid fibril structures to date, which contain steric zipper^53,56^ or zipper-like^48,49^ motifs, although these differ in length and sequence. However, of all fibrils structures solved to date only β_2_m fibrils are stabilised by a disulfide bond (Fig. 5d). Interestingly, reduced β_2_m cannot form canonical long, straight amyloid fibrils in vitro^51,57^, and reducing agents do not disaggregate preformed fibrils^27^, consistent with the buried location of the disulfide bond in the structure presented here.

Also unique to the β_2_m fibrils is the ladder of six tyrosine residues (Tyr63, Tyr66, and Tyr67 from each protofilament) that form an extended π-stack perpendicular to the fibril axis (Fig. 5c). This six ring π-stack at the interprotofilament interface is also different from the interfaces found in other fibrils, which involve steric zippers (α-synuclein^12,13^ and Aβ_40_/Aβ_42_^10^), and backbone hydrogen bonding or coordination of the disordered terminus in different polymorphs of Tau in Alzheimer’s disease (Supplementary Table 7)^11^. Although π-stacking down the fibril long axis is common in these amyloid fibrils, we know of no other examples of fibrils with perpendicular π-stacks in this manner. An analysis of the available structures in the RCSB PDB suggests that stabilisation of interchain interfaces by π-stacking is uncommon. Indeed, an analysis of 136,162 structures (Supplementary Tables 8, 9) showed that the only interchain interactions involving four or more face-to-face aromatic rings were found stacked along the fibril axis of other amyloid fibrils (Supplementary Table 9, Supplementary Figure 8b). Even intrachain, face-to-face π-stacks containing four or more rings were rare; only found running down the axes of β-helices (Supplementary Figure 8a), in an unnamed motif common to lectins (Supplementary Figure 8c), and a formyltetrahydrofolate deformylase (PDB 3nrb) which has a unique face-to-face stack made up of tyrosine and phenylalanine residues (Supplementary Figure 8d).

The molecular details of the β_2_m fibril structure presented here allow interpretation of previous mutagenesis data, particularly in light of the regions involved in stabilising the subunit structure and protofilament interface. The E-strand (residues Phe62–Phe70) of native β_2_m has long been identified as a region of high amyloidogenicity^35,58^. Accordingly, short peptides derived from this region rapidly assemble into amyloid-like fibrils while other β_2_m-derived peptides are not able to do so, or form fibrils with structures unrelated to those formed by the full-length protein^59^. These results are consistent with the central role that the E strand sequence plays in the fibril subunit structure and interprotofilament interface revealed here. For example, the F62A, Y63A, Y67A triple mutant protein is unable to form fibrils at low pH, and the L65A and F70A variants of β_2_m are severely compromised both in their ability to form fibrils and to elongate wild-type seeds^58^, presumably because they disrupt the stability of the inter-protofilament interface, as well as the core subunit structure itself. The L40R variant is poor at elongating wild-type seeds, but is able to form fibrils on its own^58^. This residue is involved in the steric zipper stabilising the subunit of the wild-type fibril, suggesting the possibility of the L40R variant forming a different fibril morphology. Finally, the natural variant D76N that forms a systemic amyloidosis of the viscera^43^ is found in the subunit core in a region stabilised by an array of hydrophobic interactions. This raises the possibility that D76N fibrils form a different structure to that presented here for the wild-type protein, consistent with MAS-NMR data of the location of β-strands in D76N fibrils formed in vitro^44^; that cross-seeding wild-type β_2_m monomers with D76N results in fibrils distinct from those formed by polymerisation of wild-type protein alone^60^ and that co-polymerisation of D76N and wild-type β_2_m does not occur in heterozygous individuals with D76N amyloidosis^43^.

Common pathways of aggregation via nucleated assembly underlie amyloid formation in a diverse range of unrelated proteins^61,62^, including more than 50 sequences involved in human amyloid disease^1^. The first glimpses of the amyloid fold family from recent MAS-NMR and cryo-EM studies have also revealed remarkable similarities in the helical, cross-β, multi-protofilament architecture of amyloid fibrils, despite wide disparities in the protein sequences involved. The β_2_m fibril described here shows several of the structural motifs found in other fibrils solved to date: a cross-β architecture, parallel in-register stacking of β-strands, two protofilaments, subunit stabilisation by hydrophobic packing, and steric zippers. However, the β_2_m fibril also contains features that are strikingly different: protofilament interfaces stabilised by inter-sidechain H-bonds, subunit stabilisation by extensive π-stacking interactions and an essential disulfide bond. We also show that different fibril polymorphs can be formed from a common subunit structure, as shown previously for different fibril structures formed from short peptide fragments of antibody light chains^63^ and transthyretin^64^. There are thus multiple mechanisms by which a sequence can achieve a stable cross-β architecture. Understanding the details of the resulting structures may provide insights into how amyloid fibril formation relates to disease^65^, as well as providing inspiration for the development of small molecules that interact with aggregation to impede or accelerate the formation of individual fibril types^66^.

## Methods

### Preparation of β_2_m fibrils

β_2_m was recombinantly-expressed in *Escherichia coli*. Samples for cryo-EM were grown in LB medium^38^, while isotopically labelled samples used for MAS-NMR were grown in M9 or HCDM1 medium with a single carbon and nitrogen source. All MAS-NMR samples used for this study were uniformly ^15^N labelled using ^15^NH_4_Cl (Cambridge Isotope Laboratory (CIL)). A total of 5 samples were generated using different carbon sources: (1) [u-^13^C, u-^15^N]-β_2_m using [^13^C_6_]-glucose, (2) [1,6-^13^C_2_, u-^15^N]-β_2_m using [1,6-^13^C_2_]-glucose, (3) [1,3-^13^C_2_, u-^15^N]-β_2_m using [1,3-^13^C_2_]-glycerol, (4) [2-^13^C_1_, u-^15^N]-β_2_m using [2-^13^C_1_]-glycerol, and (5) [^13^C-VYL, u-^15^N]-β_2_m using [^13^C, ^15^N]-VYL. Fibrils for cryo-EM were formed by dissolving purified, monomeric β_2_m in buffer containing 25 mM sodium phosphate, 25 mM sodium acetate pH 2.5, and 0.03% (w/v) NaN_3_ at 0.25 mg/mL (21 µM) β_2_m and incubating quiescently for 5 weeks at 37 °C. For MAS-NMR, de novo wild-type β_2_m fibrils were generated by re-suspending lyophilised protein in 25 mM sodium phosphate, 25 mM sodium acetate buffer at pH 2.5, containing 50 mM NaCl, 0.02 % (w/v) NaN_3_ at a concentration of 83 μΜ. Several samples of 1 mL were incubated each in 2 mL Eppendorf tubes at 37 °C with 200 rpm orbital shaking for 14 days. The samples were collated and transferred to a single tube and fibrils then pelleted by 30 min centrifugation at 14,000*g* using a bench-top centrifuge. Control experiments using MAS-NMR showed that identical spectra were obtained when fibrils were grown in the presence of 50 mM NaCl. The long, straight morphology of each fibril preparation was confirmed using EM and/or AFM.

### MAS-NMR experiments

Isotopically labelled samples were individually packed into a 3.2 mm Bruker rotor (Bruker BioSpin, Billerica, MA) using a home-built centrifugal packing tool. Typically, ∼30 mg of hydrated β_2_m fibrils were needed for a fully packed rotor.

### Chemical shift assignment

^13^C and ^15^N chemical shifts of the resonances arising from residues in the fibril core of β_2_m were assigned using multi-dimensional assignment spectra conducive to backbone sequential walks as previously published^53^. Briefly, a set of 3D NCACX, 3D NCOCX, and 3D CONCA spectra were acquired on a Cambridge Instruments 750 MHz spectrometer operating under RNMR (courtesy of Dr. David Ruben). The spectra were recorded at *ω*_r_/2*π* = 12.5 kHz and regulated to ±10 Hz using a Bruker MAS I spinning frequency controller. DARR mixing was used for the 3D NCACX (*τ*_mix_ = 60 ms) and 3D NCOCX (*τ*_mix_ = 80 ms). Additionally, 3D NNC^α^ experiments were acquired on Bruker 800 and 900 MHz AVANCE III spectrometers equipped with a 3.2 mm triple channel HCN Bruker probe (Bruker Biospin, Billerica, MA). Spectra were recorded at *ω*_r_/2*π* = 20 kHz and regulated to ±10 Hz using a Bruker MAS II spinning frequency controller. The 15 ms ^15^N–^15^N PAR mixing used radio frequency (RF) fields of *ω*_1H_/2*π* = 55.4 kHz and *ω*_15N_/2*π* = 32.2 kHz. Spectra recorded at *ω*_0H_/2*π* = 750 MHz were processed with the NMRPipe software package, while spectra recorded at *ω*_0H_/2*π* = 800 and 900 MHz were processed using TopSpin 3.2. All spectra were analysed in Sparky. ^13^C and ^15^N chemical shifts were referenced using the published shifts of adamantine relative to DSS for ^13^C referencing and the IUPAC relative frequency ratios between DSS (^13^C) and liquid ammonia (^15^N). A list of acquisition and processing parameters with additional references can be found in Supplementary Tables 10 & 11. All experiments were conducted at 268 K.

### Distance constraints from MAS-NMR spectroscopy

A total of 1157 contacts were observed, of which 399 were classified as intra-residue, 385 as sequential, 229 as medium-range, and 144 as long-range contacts. These contacts were extracted from a total of 19 2D ^13^C–^13^C and ^13^C–^15^N correlations. We recorded ^13^C–^13^C-PDSD, ^13^C–^13^C-PAR, ^13^C–^13^C-RFDR, and ^13^C–^15^N-PAIN spectra on almost all isotopically labelled samples. A full list of acquisition and processing parameters with additional references can be found in Supplementary Tables 10, 11.

### Cryo-EM grid preparation

β_2_m fibrils were diluted 1:10 with fibril buffer to a final concentration of 0.025 mg/mL (2.1 µM) monomer equivalent concentration. A 300 mesh copper EM grid with Quantifoil R3.5/1 carbon film (Electron Microscopy Services) was glow discharged in a Cressington 208 carbon evaporator fitted with a glow discharge unit for 1 min at 10 mA power. Four microlitres of the sample was applied to the grid, which was then blotted with Whatman #40 filter paper and plunge-frozen in liquid ethane using an EM-GP plunge freezer (Leica).

### EM data collection

EM images were collected using a Titan Krios (ThermoFisher) electron microscope operating at 300 keV and recorded on an energy filtered K2 direct detector (Gatan) with a pixel size of 1.06 Å/pixel. 5549 micrographs were recorded in two sets with total electron doses of 42.3 and 35.8 e^−^/Å^2^. The dose was fractionated into 40 frames for per frame doses of 1.05 and 0.89 e^−^/Å^2^ respectively. Of the 5549 micrographs collected, 2012 (~36%) contain fibrils.

### Data processing

Frames 3–40 of each micrograph movie were motion-corrected, dose weighted^67^, and merged using motioncor2^68^. The contrast transfer function (CTF) for each micrograph was determined using gCTF^69^ on motion-corrected, but non-dose weighted, micrographs.

### Helical reconstruction

All reconstruction was performed using Relion2.1^40^. As the micrographs contained multiple fibril morphologies, fibrils with a similar gross morphology were first selected by eye. The selected fibrils were segmented into 300 × 300 pixel boxes overlapping by 90% (270 px). One round of 2D classification was performed; only class averages showing an obvious β-sheet repeat (Fig. 1a—inset) were retained. An initial round of 3D classification was performed using a previous, low-resolution structure of β_2_m fibrils (EMD-1613^38^) filtered to 60 Å as a reference. Multiple rounds of 3D classification were then performed with the best model from the previous iteration filtered to 40 Å as a reference. Initial helical symmetry parameters were estimated from measurements of the fibril and refined with local symmetry searches. The possibility of the fibrils having a 2_1_ screw axis symmetry was also explored; after initial helical symmetry parameters were determined, a new set of local symmetry searches were performed using the equivalent 2_1_ screw axis helical parameters. In all cases, this resulted in lower resolution structures with obvious artefacts from the application of incorrect symmetry (Supplementary Figure 9). Exhaustive symmetry searches were performed around the initial symmetry parameters to further refine the helical symmetry. There is some anisotropy in the *Z* dimension of the reconstruction which means we cannot completely preclude the possibility of the refinement having converged to a local optimum or having slightly symmetry parameters.

When no further improvement was observed in the classes generated from 3D classification the particles that contributed to the best class were traced back to their original micrographs and those fibrils containing long runs of contributing segments were re-segmented using the original parameters. Multiple rounds of 3D classification were then again used to generate an optimised particle stack for the final refinement.

All 3D classification was performed using a *T* value^39^ of 4. A previous fibril reconstruction^11^ reported the necessity of using higher *T* values to separate fibril morphologies but this was not found to be necessary for this dataset.

One round of refinement with the helical symmetry parameters of 4.83 Å rise and −0.608° twist yielded the initial fibril map at 4.2 Å resolution. The final map was generated using the same helical parameters with an additional C_2_ symmetry applied across the fibril axis, giving a final model at 3.9 Å resolution by gold standard FSC^70^. Classification and refinement were performed using 30% for the ‘*helical_z_percentage*’ parameter^40^, and final post-processing was performed with a value of 10%.

A lower resolution reconstruction of the single protofilament fibril polymorph was made using the above methods, except a *T* value of 20 was used for 3D classification, the backtracking and re-extraction steps were omitted, and no post-processing was performed. The absolute handedness off the low-resolution reconstruction was not determined, and the fibril was assumed to be left-handed. The resolution of this reconstructions was estimated to be 6.7 Å by analysis of correlation between neighbouring Fourier pixels using the program rmeasure^71^, although the overall appearance the reconstructions, judged by the features that can be resolved suggests these resolution estimates are substantially overestimated due to the high symmetry applied.

### Model building and refinement

A single chain of β_2_m was built and manually refined using COOT^72^ and 7 copies of the resulting model fit into the EM map using UCSF Chimera^73^ to preserve nearest neighbour interactions during subsequent refinement steps. The resulting stack of 8 subunits was then subjected to multiple rounds of real space refinement with NCS restraints in Phenix^74^. Initial refinement iterations were performed with no secondary structure restraints, the results of each round of refinement were analysed with STRIDE^75^ to detect secondary structure elements. Distance restraints obtained from the MAS-NMR experiments were used to restrain backbone torsion angles during the final stages of refinement. All refinements were performed using map information to 4.0 Å resolution. For final statistics of the refined model, see Supplementary Table 12. Surface complementarity of the steric zipper within this model was calculated using the program SC using default parameters.

### Searching the PDB for π-stacking interactions

A python script was written to analyse all of the structures deposited in the RCSB PDB and search for π-stacking interactions similar to those found in the fibril structure. The ring centre for Phe and Tyr residues was defined as the point central to the six atoms that make up the ring (CG, CD1, CD2, CE1, CE2, and CZ) and the ring centre for Trp was defined as the point central to atoms CD2 and CE2. A ‘face-normal’ vector was used to define the orientation of each ring, calculated as the cross product of the centre to CG and centre to CD1 vectors for Phe/Tyr and centre to CD1 and centre to CD2 for Trp (Supplementary Figure 10). Two residues were defined to have a face-to-face π-stacking interaction if the centre-to-centre distance was less the 6 Å and difference between face normal vectors 0 ± 20° or 180 ± 20°. The script was then run on all of the structures deposited in the PDB, downloaded on the 17th of January, 2018. The resulting π-stack interactions predicted to involve greater than 5 rings were manually examined and curated.

### Scanning electron microscopy

β_2_m fibrils were adsorbed onto a copper EM grid coated with a layer of amorphous carbon and lightly stained with 1% (w/v) uranyl acetate. The fibril morphology was confirmed using transmission electron microscopy (TEM) and then SEM imaging conducted with a Hitachi SU-8230 cold field emission scanning electron microscope (CFE-SEM) operated at 3.5 keV with beam deceleration of 1.5 keV. Images were recorded at a nominal magnification of 250,000× yielding a pixel size of 3.9 Å/px.

### Fibril sample preparation for AFM studies

Fibril seeds were prepared by stirring a sample (500 μL) of lyophilised wild-type β_2_m re-suspended in a buffer of 10 mM sodium phosphate pH 2.0 containing 50 mM NaCl at a concentration of 120 μΜ, in a 1.5 mL glass vial containing a PTFE magnetic stirrer. Stirring was performed in a custom made magnetic stirrer built by the Department of Physics and Astronomy (University of Leeds) at 1000 rpm in room temperature for 2 days. To monitor the time course of fibril elongation, lyophilised wild-type β_2_m was re-suspended in the same buffer at a concentration of 120 μΜ with 10% (v/v) of seeds added. At various timepoints during fibril elongation, fibrils were deposited on mica surfaces. To ensure uniform coverage and dispersion of the entire surface, the sample was diluted to 0.4 μΜ with freshly made sterile filtered deionised water. A drop of 20 μL sample was deposited on the mica surface followed by incubation for 5 min. The surface was washed by pipetting quickly 1 mL of sterile water and was then immediately dried by applying a gentle steam of N_2_ gas.

### Atomic force microscopy

Tapping-mode atomic force microscopy was performed utilising a Dimension 3100 Scanning Probe Microscope (Veeco Instruments) and PPP-NCLR silicon cantilever probes (Nanosensors, Neuchatel, Switzerland). The images collected were 10 × 10 μm in each dimension (1024 × 1024 pixels) in height trace mode (scan rate was constant at 0.80 Hz). Acquisition of the image was followed by processing using the NanoScope 6.13rl software to remove surface tilt and scanner bow (by application of a 3rd order polynomial planefit).

### Analysis of AFM images

AFM images were analysed using scripts^61^ written in MATLAB (Mathworks). Briefly, only fibrils that do not interfere with the image boundaries, do not overlap with neighbouring fibrils, are 4–7 pixels in width and at least 4 pixels in length were unambiguously traced. The length distribution that is biased towards shorter fibrils was then bias-corrected^76^. A Weibull probability density function, found to best describe such a distribution was applied to fit the bias-corrected data and thus length parameters could be calculated. At least 300 fibrils were analysed for each elongation time-point.

### Code availability

The script for analysis of PDB files for π-stacking interactions is available at https://github.com/attamatti/findpi.

## Electronic supplementary material

Supplementary Information

Description of Additional Supplementary Files

Supplementary Movie 1

Supplementary Movie 2

## Data Availability

The structures of the single and double protofilament β_2_m amyloid structures were deposited in the Electron Microscopy Data Bank with accession numbers EMD-0021 and EMD-0014, respectively. In addition, the atomic model built into the 3.9 Å double protofilament reconstruction (EMD-0014) was deposited in the Protein Data Bank with the PDB-ID 6gk3. The raw cryo-EM data has been deposited in the EMPIAR database (EMPIAR-10207). All data that support the findings of this study are available from the corresponding authors upon reasonable request.
