# Supplementary material for: The structure of a β2-microglobulin fibril suggests a molecular basis for its amyloid polymorphism
Source: Nat Commun. 2018 Oct 30;9:4517. doi: 10.1038/s41467-018-06761-6 (PMC6207761; doi:10.1038/s41467-018-06761-6)
Supplement: Supplementary file 1 — Supplementary Information [file 41467_2018_6761_MOESM1_ESM.pdf]

Supplementary Information:

Iadanza et al, (2018). The structure of a β2m fibril suggests a molecular basis for its amyloid polymorphism

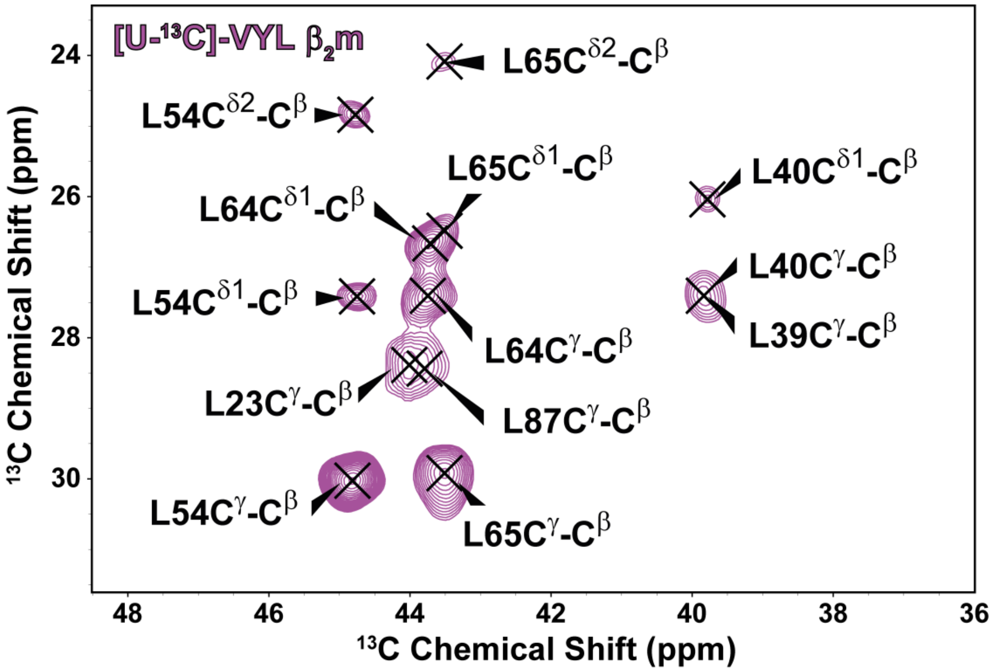

Supplementary Figure 1 MAS-NMR of β2m amyloid fibrils. *The region of a short-mixing time 2D ^{13}C-^{13}C-RFDR spectrum showing short-distance C^{β}-C^{γ} correlations and medium-distance C^{β}-C^{δ} correlations of a [^{13}C]-VYL labelled sample shows no peak doubling, reinforcing the conclusion that the subunit making up the fibril core is monomorphic on a molecular level.*

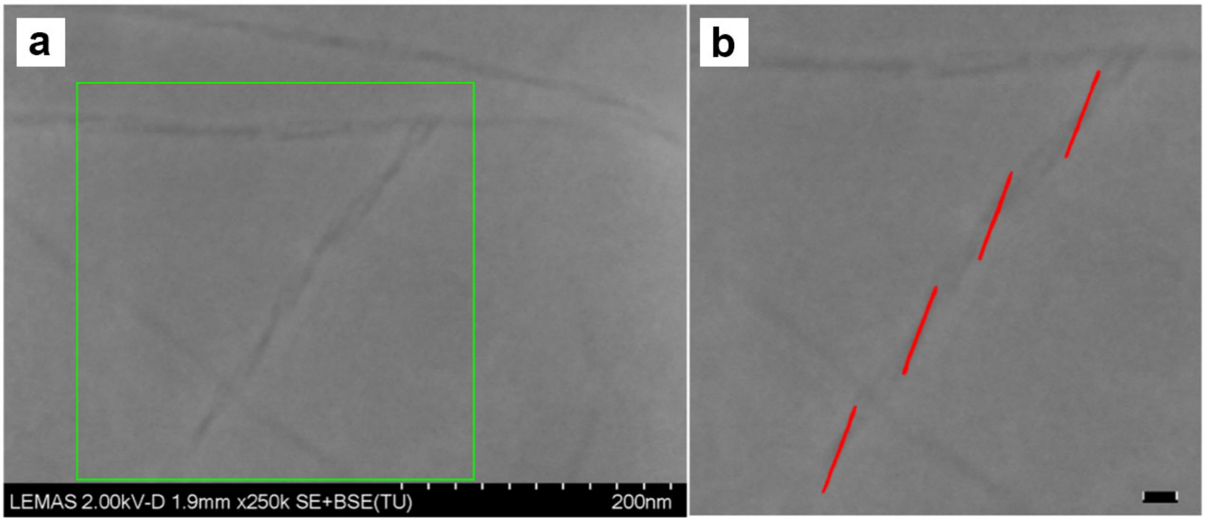

Supplementary Figure 2. Scanning electron microscopy (SEM) images of fibrils verify the left-handed twist. *(A) SEM image of a two-protofilament fibril (type C in Figure 6 main text). (B) Zoomed in image for the green boxed area in panel A. Red lines trace the left-handed crossovers. Scale bar is 20 nm.*

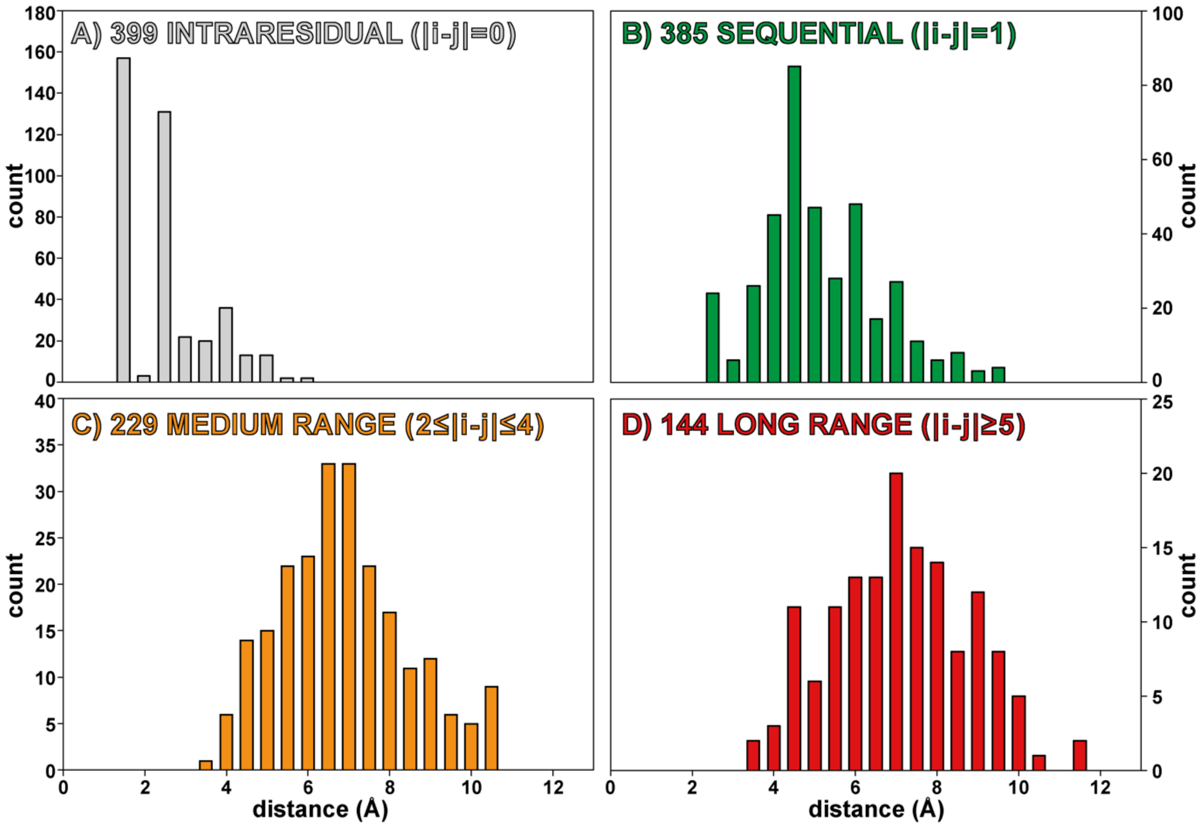

Supplementary Figure 3. Histogram of contact distances observed by MAS NMR spectroscopy. *A total of 1157 contacts were observed, of which (A) 399 were classified as intra-residual, (B) 385 as sequential, (C) 229 as medium-range, and (D) 144 as long-range contacts. Distances were binned at a 0.5 Å step size.*

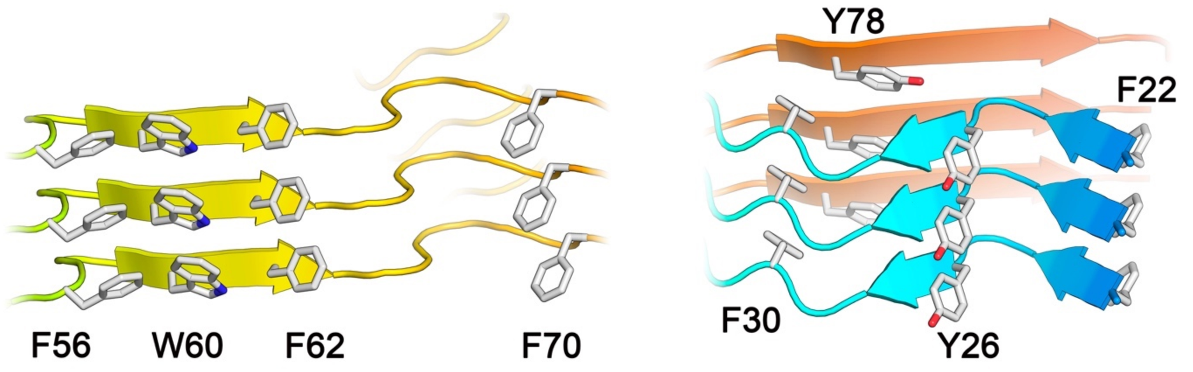

Supplementary Figure 4. Canonical π-stacking stabilises the fibril structure. The canonical parallel in-register cross-β structure is maintained down the fibril long axis is supported by extensive π-stacking interactions between the aromatic residues F22, Y26, F30, F56, W60, F62, F70, and Y78 along the length of the core.

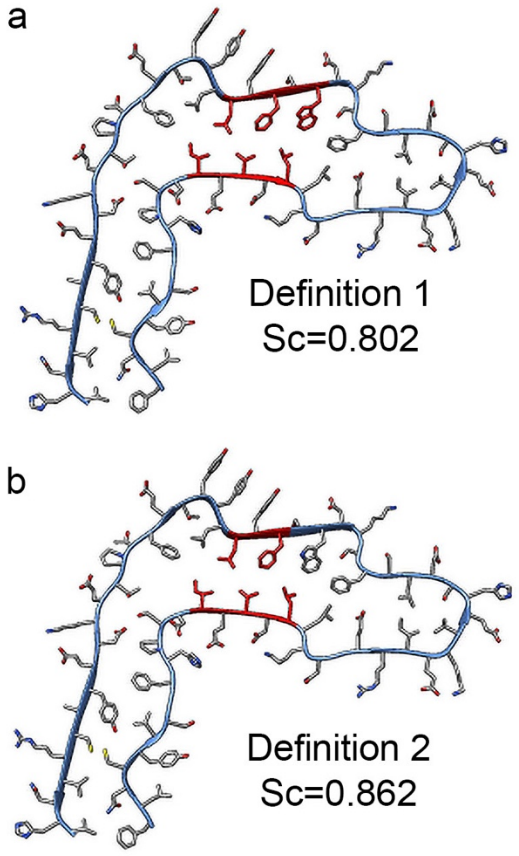

Supplementary Figure 5. Surface complementarity calculations. *Surface complementarity was calculated using the program SC[1], including either 6 residues (a) or 5 residues (b) in the zipper (shown in red).*

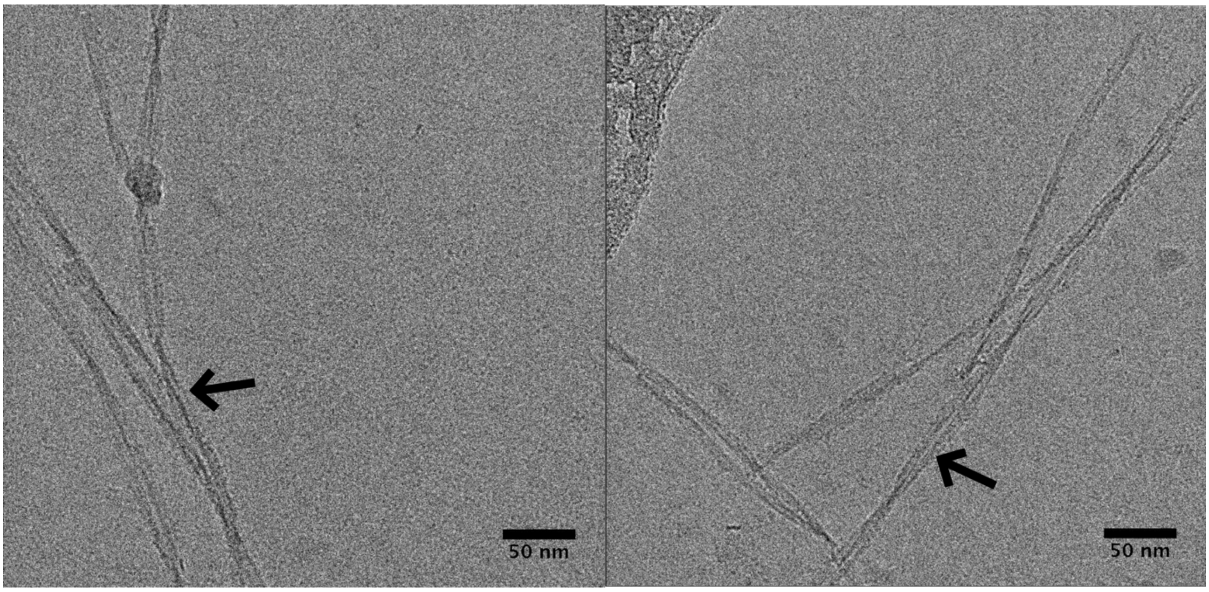

Supplementary Figure 6. Higher order fibril assemblies. *Cryo-EM images of β_2m fibrils that appear to be composed of multiple copies of a common underlying fibril structure. Fibril morphologies twisting together are marked with arrows.*

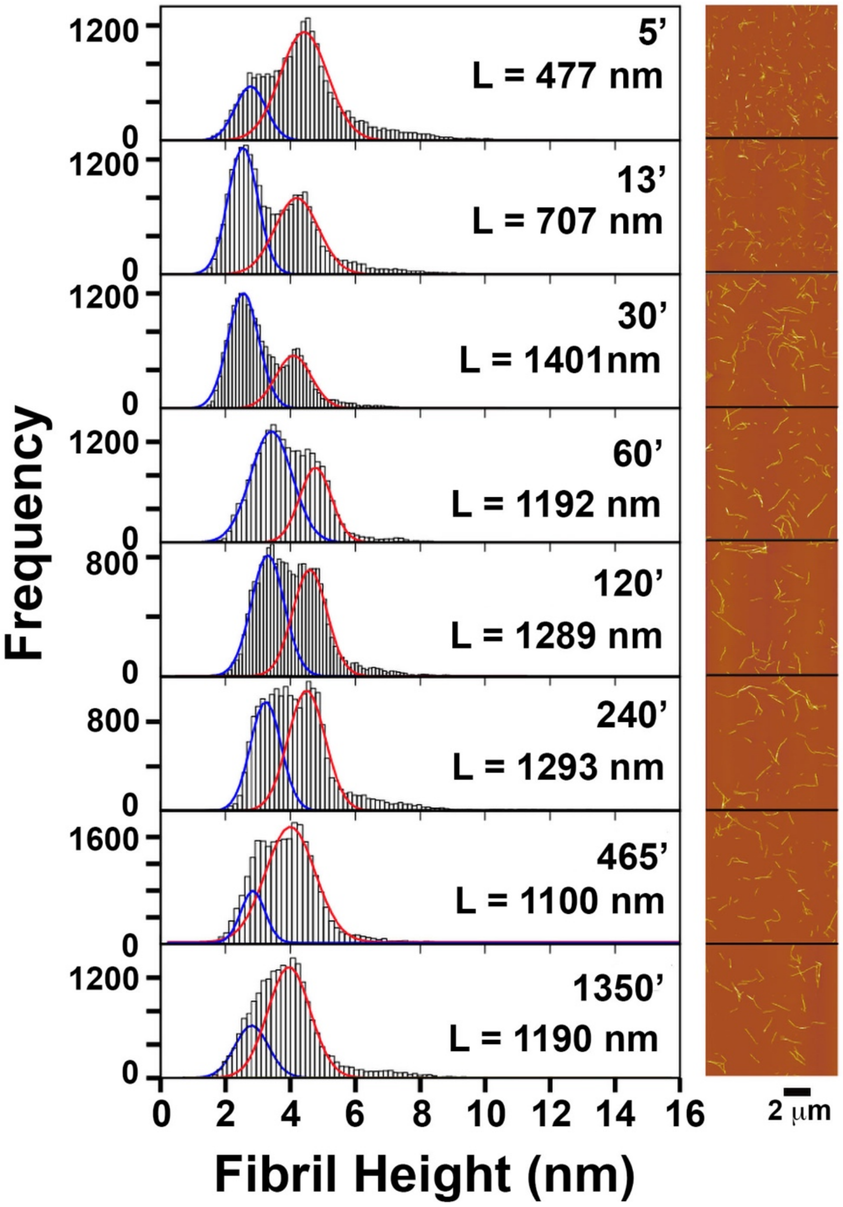

Supplementary Figure 7. Single protofilament fibrils are precursors of higher order assemblies. *Histograms of fibril height were measured by AFM at various time-points after seeding was initiated. The fibril length (L) is indicated in each plot. Representative AFM images are shown alongside. The red and blue lines represent fits to (two) Gaussian functions. These real-time studies of fibril height show that while both thin and thick filaments are formed rapidly in assembly, at early times thin fibrils are more highly populated, suggesting that these thicken during assembly via protofilament-protofilament interactions to create the array of polymorphs shown in Figure 6 (main text).*

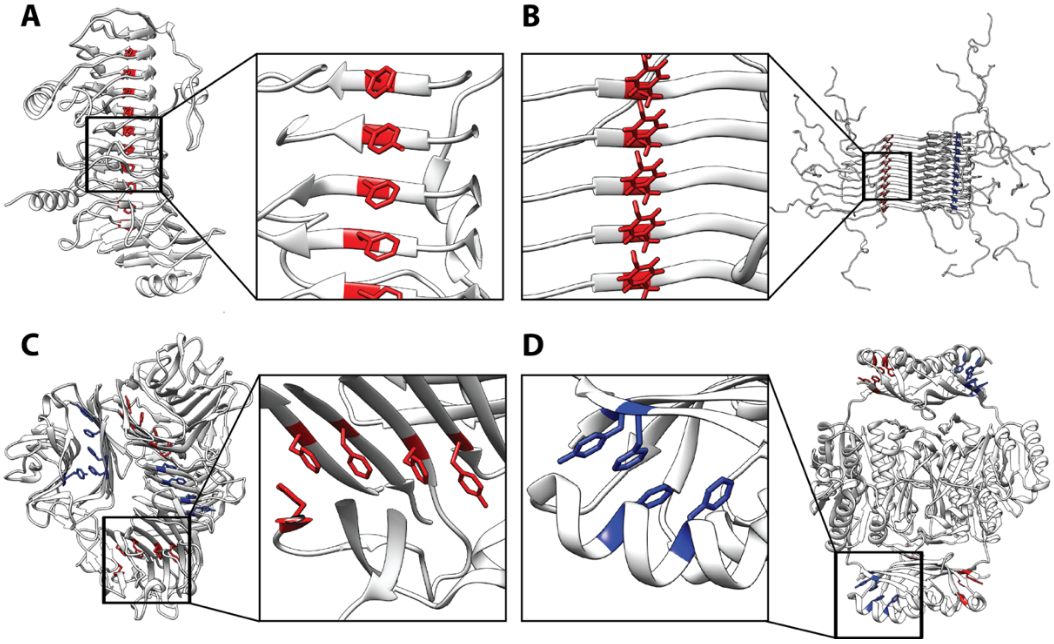

Supplementary Figure 8. Examples of types multi-ring π-stacking interactions identified in the pdb. (*a) Parallel aromatic rings down the axis of a β-helix, illustrated using chondritonase B (pdb 1ofm)[2]. (b) Parallel to the axis of an amyloid fibril, illustrated using the MAS NMR structure of an α-synuclein fibril (pdb 2n0a)[3]. (c) A ring-stack motif common to lectins, illustrated using peanut lectin (pdb 1cq9)[4]. (d) A unique ring stack motif found in Psuedomonas putida formyltetrahydrofolate deformylase (pdb 3nrb).*

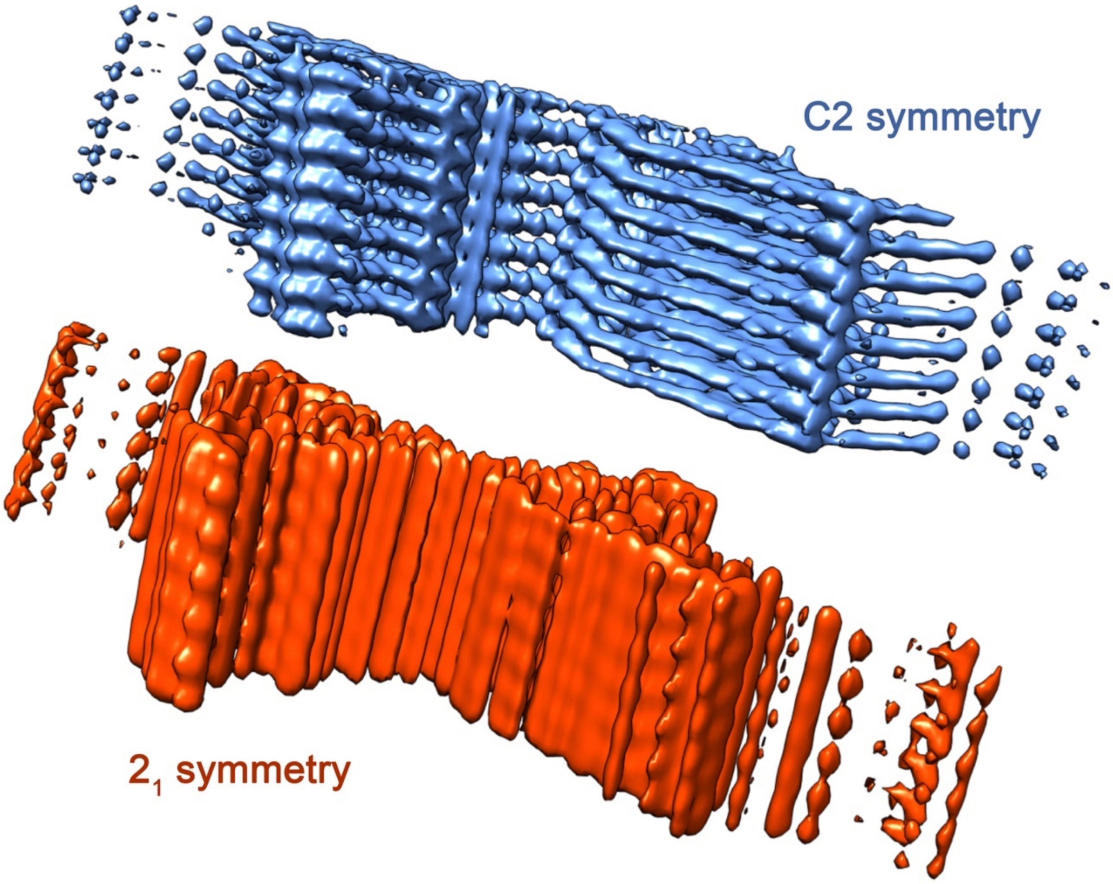

Supplementary Figure 9. The symmetry of the two protofilament β_2m fibril. *Extensive trials of different symmetries were tested. Of particular interest were symmetries with either C2 or 2_1 screw axes, In an object with a 2_1 screw axis, the subunit in the two protofilaments are related by a 180º rotation and a translation, resulting in the two protofilaments being either in-register (C2) or out of register (2_1). This is exemplified in the two conformers of Tau published recently[5], where Tau PHFs have a 2_1 screw symmetry, but Tau SF do not. For β_2m, application of a 2_1 screw symmetry leads to loss of separation of the β-strands, and poorer resolution.*

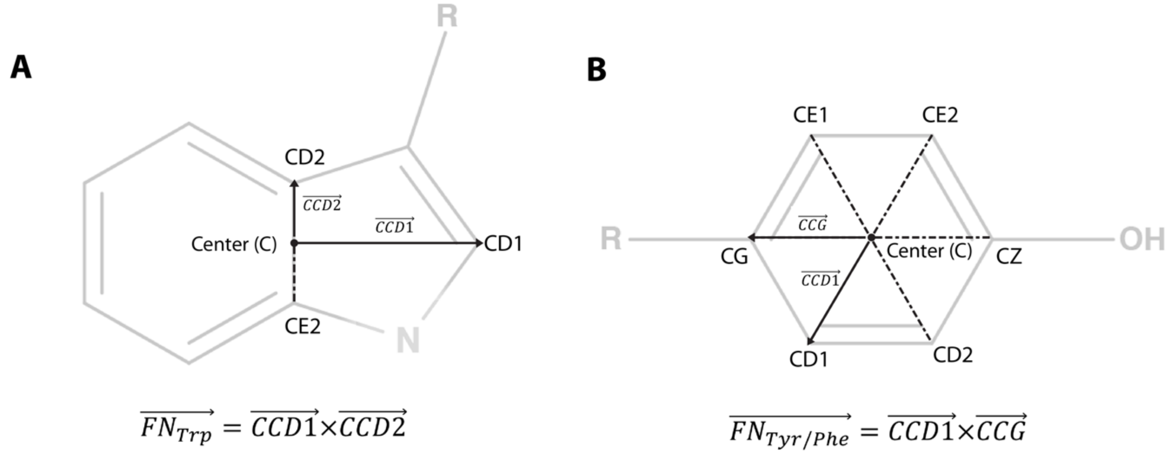

Supplementary Figure 10. Parameters for defining face-to-face π-stacking interactions in the PDB. *Two aromatic rings were considered to be in a π-stack if the distance between centres was 6 Å or less and the angle between the two face-normal (FN) vectors (A & B) was 0˚± 20˚ or 180 ± 20˚.*

Supplementary Table 1: Breakdown of the different fibril morphologies observed and their contribution to the two protofilament structure (labelled C in Figure 6, main text).

Fibril polymorph	A	B	C	D	E	F	Multiple fibril aggregates
Number observed	132	884	1737	128	186	38	2590
% non-aggregated fibrils	4.2	28.4	55.9	4.1	5.9	1.2	n/a
Number in reconstruction	0	0	622	0	0	0	0
% in reconstruction	0	0	35	0	0	0	0

Supplementary Table 2: Inter-nuclear contacts between atoms within an amino acid residue observed by MAS-NMR spectroscopy.

1st nucleus	2nd nucleus
F22CA	F22CG
L23CD1	L23CB
L23CA	L23C
L23CB	L23CA
L23CD1	L23CA
L23CD1	L23CG
L23CD1	L23C
L23CD2	L23CA
L23CD2	L23CB
L23CD2	L23CD1
L23CD2	L23CG
L23CG	L23CA
L23CG	L23CB
L23CG	L23C
L23N	L23CA
L23N	L23CD2
N24CB	N24CA
N24ND2	N24CB
C25CB	C25CA
C25CB	C25C
C25N	C25CB
Y26CA	Y26CZ
Y26CB	Y26CG
Y26CB	Y26CZ
Y26CB	Y26CA
Y26CA	Y26C
Y26CA	Y26CE1
Y26CA	Y26CG
Y26CB	Y26CE1
V27CA	V27C
V27CB	V27CA
V27CB	V27C
V27CG1	V27CA
V27CG1	V27C
V27CG2	V27CA
V27CG2	V27CB
V27CG2	V27CG1
V27CG2	V27C
V27N	V27CA
V27N	V27CG1
V27N	V27CG2
S28CA	S28CB
S28N	S28CB
G29N	G29CA
F30CA	F30CG
F30CB	F30CG
F30CB	F30CA
F30CE1	F30CG
F30CB	F30CD2
H31CA	H31CE1
H31CA	H31C
H31CB	H31CD2
H31CB	H31CG

1st nucleus	2nd nucleus
H31CB	H31C
H31CB	H31CA
H31CA	H31CG
H31CB	H31CE1
P32CB	P32CA
P32CB	P32CD
P32CG	P32CA
P32CG	P32CD
P32CD	P32CA
P32N	P32CA
P32N	P32CG
P32CA	P32C
P32CB	P32C
P32CG	P32CB
P32CG	P32C
S33N	S33CA
S33N	S33CB
D34CB	D34CA
I35CD1	I35CA
I35CD1	I35CB
I35CD1	I35CG1
I35CD1	I35CG2
I35CG1	I35CA
I35CG1	I35CB
I35CG2	I35CA
I35CG2	I35CB
I35CD1	I35C
I35CB	I35CA
I35N	I35CA
I35N	I35CB
I35N	I35CD1
I35N	I35CG1
I35N	I35CG2
I35CG2	I35CG1
I35CG2	I35C
E36CG	E36CD
V37CG2	V37CA
V37CG2	V37CB
V37CG2	V37CG1
V37N	V37CG2
V37CG1	V37CB
L39CA	L39C
L39CB	L39CA
L39CB	L39C
L39CD1	L39CA
L39CD1	L39C
L39CD2	L39CA
L39CD2	L39CD1
L39CG	L39CB
L39CD2	L39CB
L39CD1	L39CB
L40CB	L40CA
L40CD1	L40CB

1st nucleus	2nd nucleus
L40CG	L40CB
L40CD1	L40CA
L40CA	L40C
L40CB	L40C
L40CD1	L40CG
L40CD1	L40C
L40CG	L40CA
L40CG	L40C
L40N	L40CA
L40N	L40CB
L40N	L40CD1
L40N	L40CG
K41NZ	K41CE
K41CD	K41CE
K41CD	K41CA
K41CA	K41C
N42CB	N42CA
N42CB	N42CG
N42N	N42CA
N42N	N42CB
N42ND2	N42CA
N42ND2	N42CB
G43CA	G43C
G43N	G43CA
E44CB	E44CA
E44N	E44CB
E44N	E44CG
E44CB	E44CD
R45CB	R45CD
R45CG	R45CB
R45CG	R45CZ
R45CG	R45CA
R45CG	R45CD
I46CB	I46CA
I46CD1	I46CA
I46CD1	I46CB
I46CD1	I46CG1
I46CD1	I46CG2
I46CG1	I46CA
I46CG1	I46CB
I46CG2	I46CA
I46CG2	I46CB
I46CG2	I46CG1
I46CD1	I46C
I46N	I46CA
I46N	I46CD1
I46N	I46CG1
I46N	I46CG2
K48CB	K48CD
K48CB	K48CE
K48CD	K48CE
K48CG	K48CE
K48CB	K48CA

1st nucleus	2nd nucleus
K48CD	K48CA
K48CE	K48CA
K48CG	K48CA
K48CG	K48CB
K48CG	K48CD
K48N	K48CB
V49CG1	V49CB
V49CG1	V49CA
V49CA	V49CB
V49CB	V49C
V49CG1	V49C
V49N	V49CA
V49N	V49CB
H51CB	H51CA
H51CB	H51CE1
H51CB	H51CG
H51CA	H51CG
H51CA	H51CD2
H51CB	H51CD2
H51CD2	H51CE1
S52CA	S52CB
S52CB	S52C
S52N	S52CA
S52N	S52CB
D53CB	D53CA
L54CA	L54CB
L54CD2	L54CB
L54CG	L54CB
L54CA	L54CD1
L54CA	L54CD2
L54CA	L54CG
L54CB	L54CD1
L54CD1	L54CG
L54CD2	L54CD1
L54CD2	L54CG
L54CA	L54C
L54CB	L54C
L54N	L54CB
L54N	L54CD1
L54N	L54CD2
L54N	L54CG
S55CA	S55CB
S55N	S55CA
F56CB	F56CA
F56CA	F56CG
F56CB	F56CG
F56CB	F56C
F56CA	F56CE1
F56CE1	F56CG
F56N	F56CB
F56CB	F56CD1
S57CA	S57CB
S57N	S57CA

1st nucleus	2nd nucleus
K58CD	K58CB
K58CB	K58CA
K58CD	K58CA
K58CG	K58CA
K58CG	K58CB
K58CG	K58CD
D59CB	D59CA
D59N	D59CB
W60N	W60CG
W60CA	W60CG
W60CA	W60CD1
W60CA	W60CZ2
W60CB	W60CA
W60CB	W60CD1
W60CB	W60CG
W60CB	W60CZ2
W60CD1	W60CE2
W60CD1	W60CG
W60CA	W60CD2
W60CA	W60CH2
W60CB	W60CD2
W60CB	W60CH2
W60CG	W60CD2
W60CG	W60CH2
W60CG	W60CZ2
W60CH2	W60CD1
W60CH2	W60CD2
W60CH2	W60CE2
W60CZ2	W60CD2
W60CZ2	W60CH2
W60N	W60CA
W60NE1	W60CD1
W60CA	W60CE3
W60CB	W60CE2
W60CB	W60CE3
W60CE3	W60CE2
W60CA	W60CE2
W60CD1	W60CD2
W60CD1	W60CE3
W60CD2	W60CE2
W60CD2	W60CE3
W60CE2	W60C
W60NE1	W60CE2
S61CA	S61CB
S61CA	S61C
S61CB	S61C
F62CA	F62CB
F62CB	F62CG
F62CA	F62CG
F62CZ	F62CD1
F62CZ	F62CD2
F62N	F62CB
F62CB	F62CD1

1st nucleus	2nd nucleus
Y63CA	Y63CG
Y63CA	Y63CZ
Y63CB	Y63CA
Y63CB	Y63CG
L64CD1	L64CB
L64CB	L64CA
L64CA	L64CD2
L64CA	L64CG
L64CD2	L64CB
L64CD2	L64CG
L64CG	L64CB
L64CA	L64C
L64CD1	L64CA
L64N	L64CA
L64N	L64CB
L64N	L64CD2
L65CD2	L65CB
L65CG	L65CB
L65CA	L65CB
L65CA	L65CD1
L65CA	L65CD2
L65CD1	L65CG
L65CD2	L65CD1
L65CD2	L65CG
L65CA	L65C
L65CD1	L65CB
L65N	L65CA
L65N	L65CB
L65N	L65CD1
L65N	L65CD2
L65N	L65CG
Y67CA	Y67CZ
Y67CB	Y67CE1
Y67CB	Y67CZ
Y67CE1	Y67CZ
T68CA	T68CB
T68CG2	T68CA
T68CG2	T68CB
T68N	T68CB
E69CB	E69CA
F70CB	F70CA
F70CA	F70CG
F70CA	F70CD2
F70CA	F70CE1
F70CD2	F70CE1
T71CA	T71CB
T71CG2	T71CB
T71CG2	T71CA
P72CB	P72CA
P72CG	P72CA
P72CD	P72CA
P72N	P72CA
P72N	P72CB

1st nucleus	2nd nucleus
P72N	P72CG
T73CA	T73CB
T73CG2	T73CA
T73CG2	T73CB
T73CB	T73C
T73N	T73CB
E74CB	E74CG
E74CA	E74CD
E74N	E74CG
E74CG	E74CA
E74CB	E74CA
K75N	K75CD
K75CD	K75CA
K75CG	K75CA
K75CG	K75CB
K75CG	K75CD
K75CB	K75CA
K75CG	K75CE
K75CB	K75CE
K75CD	K75CE
K75CD	K75CB
D76CB	D76CA
D76CB	D76CG
Y78CA	Y78CZ
Y78CD2	Y78CZ
Y78CE1	Y78CZ
Y78CB	Y78CA
Y78CA	Y78CD1
Y78CA	Y78CD2
Y78CA	Y78CE1
Y78CA	Y78CG
Y78CA	Y78C
Y78CB	Y78CD1
Y78CB	Y78CD2
Y78CB	Y78CE1
Y78CB	Y78CG
Y78CD1	Y78C
Y78CD2	Y78C
Y78CE1	Y78C
Y78CG	Y78C
Y78CZ	Y78C
Y78CE1	Y78CG
Y78CE1	Y78CD1
Y78CE1	Y78CD2
A79CB	A79CA
A79N	A79CA
A79N	A79CB
C80CB	C80CA
C80N	C80CB
R81NE	R81CB
R81NE	R81CD
R81NE	R81CG
R81NH1	R81CZ

1st nucleus	2nd nucleus
R81CB	R81CD
R81CG	R81CB
R81CG	R81CD
R81CD	R81CZ
R81CB	R81CA
R81CD	R81CA
R81CG	R81CA
V82CG1	V82CA
V82CG1	V82CB
V82CG2	V82CA
V82CG2	V82CB
V82CA	V82CB
V82CG1	V82CG2
V82N	V82CA
V82N	V82CG1
V82N	V82CG2
N83CB	N83CA
H84CB	H84CG
H84CB	H84CA
H84CA	H84CD2
H84CB	H84CD2
H84CD2	H84CG
V85CG1	V85CB
V85CG2	V85CB
V85CG1	V85CA
V85CG2	V85CA
V85N	V85CG1
V85N	V85CG2

Supplementary Table 3: Inter-nuclear contacts between atoms within adjacent (in primary sequence) amino acid residues observed by MAS-NMR spectroscopy. Contacts coloured GREEN in Figure 3e.

1st nucleus	2nd nucleus
F22CG	L23CB
F22CG	L23CG
L23CA	N24N
L23CA	N24CB
N24CA	C25CB
N24CA	C25CA
N24CB	C25CB
C25N	Y26CA
C25CB	Y26N
C25CA	Y26CB
Y26CA	V27CA
Y26CA	V27C
Y26CB	V27CA
Y26CB	V27C
Y26CA	V27CG1
Y26CA	V27CG2
Y26N	V27CA
Y26CB	V27N
Y26CE1	V27CA
Y26CG	V27CA
Y26CE1	V27CB
V27CA	S28N
V27CA	S28CA
S28CA	G29CA
S28CB	G29CA
S28C	G29CA
S28CB	G29N
G29CA	F30CA
G29CA	F30CB
G29CA	F30C
G29CA	F30CG
F30CA	H31C
F30CB	H31CD2
F30CB	H31C
F30C	H31C
F30CA	H31CG
F30CA	H31CA
F30CG	H31CA
F30CG	H31CG
F30CB	H31CB
F30CD2	H31CB
H31CB	P32CA
H31CA	P32CD
H31C	P32CA
H31CG	P32CB
H31C	P32CB
H31CD2	P32CD
H31C	P32CD
H31CD2	P32CG
H31CG	P32CG
H31C	P32CG
H31C	P32C
H31CA	P32CB

1st nucleus	2nd nucleus
H31CA	P32CA
H31CG	P32CD
H31CE1	P32CA
P32CD	S33CB
P32CA	S33CA
P32CD	S33CA
P32CB	S33CA
P32CA	S33CB
P32CA	S33N
P32CB	S33N
P32CD	S33N
P32CG	S33N
P32CB	S33CB
P32C	S33CB
S33CA	D34CA
S33N	D34CB
D34CA	I35CD1
D34CA	I35CG1
D34CA	I35CG2
D34CA	I35N
D34CB	I35CG1
D34CB	I35CG2
D34CA	I35CA
I35CD1	E36CA
I35CG2	E36CA
I35CB	E36CA
I35CA	E36N
I35CD1	E36N
I35CG1	E36N
I35CG2	E36N
I35CA	E36CA
I35CA	E36CB
E36CG	V37CB
E36CA	V37CB
V37CG2	D38CA
V37CG1	D38CA
D38CA	L39CG
D38CA	L39CB
L39CA	L40CA
L39CA	L40C
L39CD2	L40CA
L39C	L40CA
L39CA	L40CG
L39C	L40C
L39CA	L40N
L39CD2	L40N
L39CB	L40CA
L40CB	K41CG
K41CE	N42CB
K41C	N42CB
K41CG	N42N
K41CA	N42CA
K41CA	N42CB

1st nucleus	2nd nucleus
N42CA	G43CA
N42CB	G43CA
N42CB	G43C
N42CG	G43CA
N42CA	G43N
N42CB	G43N
G43CA	E44CB
G43CA	E44CD
G43CA	E44C
G43CA	E44CA
G43N	E44CA
G43N	E44CB
G43N	E44CG
G43CA	E44N
G43CA	E44CG
E44N	R45CG
R45CA	I46CA
R45CA	I46CD1
R45CB	I46CD1
R45CA	I46CG2
R45CB	I46CG2
R45C	I46CD1
R45CA	I46CB
R45CB	I46CA
R45CG	I46CB
R45CA	I46CG1
R45CD	I46CG1
I46CB	E47CA
I46CD1	E47CA
I46CD1	E47CG
I46CG1	E47CG
I46CG2	E47CA
I46CG2	E47CG
I46CA	E47CB
I46CB	E47CB
I46CG2	E47N
I46CD1	E47CB
I46CG1	E47CA
I46CG2	E47CB
I46CA	E47CA
I46CG1	E47CB
E47CA	K48CA
E47CB	K48CE
E47CA	K48CG
E47CB	K48CG
E47CB	K48CA
E47CA	K48CB
E47CA	K48CD
E47CB	K48CB
E47CB	K48CD
K48CA	V49CA
K48CB	V49CA
K48CB	V49CB

1st nucleus	2nd nucleus
K48CD	V49CB
K48CB	V49CG1
K48CG	V49CB
K48CA	V49CG1
K48CD	V49CG1
V49CG1	E50CA
V49CG1	E50CB
V49CA	E50CA
V49CB	E50CB
V49CB	E50CA
E50CA	H51CA
E50CB	H51CA
E50CB	H51CB
E50CA	H51CG
E50C	H51CA
E50CA	H51CD2
E50CB	H51CD2
E50CA	H51CE1
E50CB	H51CE1
E50C	H51C
H51CA	S52CA
H51CA	S52CB
H51CB	S52CB
H51CG	S52CB
H51CA	S52C
H51CG	S52CA
H51CA	S52N
H51CB	S52N
H51C	S52CA
H51CD2	S52CB
H51C	S52CB
H51C	S52C
S52CA	D53CA
S52CB	D53CA
S52CA	D53CB
S52CB	D53CB
S52N	D53CA
S52CB	D53N
D53CA	L54CB
D53CB	L54CB
D53N	L54CD2
D53CA	L54CG
D53CA	L54CD1
D53CA	L54CD2
D53CA	L54CA
D53CB	L54CA
D53CB	L54CD1
D53CB	L54CD2
L54CB	S55CB
L54CD1	S55CB
L54CA	S55N
L54CB	S55N
L54CD1	S55N

1st nucleus	2nd nucleus
L54CD2	S55N
L54CG	S55N
L54CB	S55CA
L54C	S55CB
L54CA	S55CB
L54CA	S55CA
S55CA	F56CA
S55CB	F56CA
S55CA	F56CB
S55CB	F56CB
S55C	F56CB
S55CA	F56CG
S55CB	F56CG
F56CB	S57CA
F56CB	S57CB
F56CB	S57C
F56CA	S57CA
F56CE1	S57CA
F56CG	S57CA
F56CB	S57N
F56CA	S57CB
S57CB	K58CA
S57CB	K58CB
S57CB	K58CD
S57CA	K58CA
S57CA	K58CG
S57CB	K58CG
K58CG	D59CA
K58CA	D59CA
K58CA	D59N
K58CB	D59CA
K58CD	D59CA
K58CA	D59CB
D59CA	W60N
D59CB	W60N
D59CA	W60CD1
D59CA	W60CE2
D59CA	W60CE3
D59CB	W60CD1
D59CB	W60CE2
W60CA	S61CB
W60CG	S61CA
W60CD1	S61CB
W60CE2	S61CB
W60CA	S61C
W60CD2	S61CA
W60CH2	S61CA
W60N	S61CB
W60CE3	S61CB
W60CB	S61CB
W60CE2	S61CA
S61CA	F62CB
S61CB	F62CB

1st nucleus	2nd nucleus
S61CA	F62CA
S61CB	F62CA
S61CB	F62CE1
S61C	F62CA
S61CA	F62CD2
S61CA	F62CG
S61CB	F62CD2
F62CB	Y63CA
F62CA	Y63CG
F62CG	Y63CA
F62CB	Y63CB
Y63CA	L64CA
Y63CB	L64CA
Y63CB	L64CB
Y63CG	L64C
Y63CG	L64CA
Y63CZ	L64CA
Y63CB	L64CD1
Y63CG	L64CD2
Y63CB	L64CG
Y63N	L64CA
L64CA	L65CD2
L64CA	L65CA
L64CG	L65CD2
L64CA	L65C
L64CA	L65CG
L64N	L65CA
L64CA	L65N
L64CG	L65N
L64CA	L65CB
L65CA	Y66CA
L65CD1	Y66CB
L65CG	Y66CB
L65CA	Y66CB
L65CB	Y66CB
Y66CB	Y67CB
Y67CA	T68CB
Y67C	T68CB
Y67CB	T68CB
T68CB	E69C
T68CB	E69CA
T68CB	E69CG
F70CA	T71CB
F70CB	T71CB
F70CD2	T71CA
T71CA	P72CA
T71CB	P72CB
T71CB	P72CD
T71CB	P72CG
T71CA	P72CB
P72CB	T73CB
P72CG	T73CB
P72CA	T73CG2

1st nucleus	2nd nucleus
P72CA	T73CB
T73CG2	E74CG
T73CB	E74CA
T73CB	E74CG
T73CB	E74CB
T73CB	E74N
T73CA	E74CA
T73CA	E74CG
E74CA	K75CA
E74CG	K75CG
E74CB	K75CA
E74CG	K75CB
E74CA	K75CB
E74CA	K75CG
K75CB	D76CA
K75CD	D76CA
K75CG	D76CA
K75CD	D76CB
K75CB	D76CB
K75CA	D76CA
K75CA	D76CB
D76CB	E77CA
D76CA	E77CG
D76CB	E77CG
D76CA	E77N
D76CA	E77CB
D76CB	E77CB
E77N	Y78CB
E77CB	Y78CD1
E77CA	Y78CD1
Y78CD2	A79CA
Y78CE1	A79CA
Y78CZ	A79CA
Y78CA	A79N
Y78CB	A79N
Y78CG	A79CA
Y78CD1	A79CB
A79CA	C80CA
A79CA	C80CB
A79N	C80CA
A79N	C80CB
A79CA	C80N
A79CB	C80CB
C80CA	R81CA
C80CB	R81CD
C80CA	R81CD
C80CB	R81CA
C80CB	R81CB
R81CA	V82CA
R81CG	V82CA
R81CD	V82CG1
R81CD	V82CG2
R81CB	V82CG1

1st nucleus	2nd nucleus
R81CA	V82CG1
R81CA	V82CG2
R81CB	V82CG2
V82CA	N83N
V82CG1	N83CA
V82CG2	N83CA
N83CB	H84CG
N83CA	H84CD2
N83CB	H84CD2
N83CA	H84CB
N83CA	H84CE1
H84CB	V85CA
H84CD2	V85CG1
H84CE1	V85CB

Supplementary Table 4: Inter-nuclear contacts between atoms within amino acid residues that are near in the primary sequence (within 2-4 residues), observed by MAS-NMR spectroscopy. Contacts coloured ORANGE in Figure 3e.

1st nucleus	2nd nucleus
L23CB	Y26CZ
L23CD2	Y26CZ
L23CA	Y26CA
L23CA	Y26CE1
L23CB	Y26CG
L23CA	C25CB
N24CB	Y26CG
C25CB	V27CG2
Y26CG	S28CA
V27C	G29CA
V27CG1	F30CD2
G29CA	H31CA
G29CA	P32CD
F30CB	P32CA
F30CG	P32CB
F30CG	P32CD
F30CG	P32CG
F30CA	P32CA
F30CG	P32CA
F30CA	P32CB
F30CA	P32CD
F30CE1	P32CG
F30CA	D34CB
F30CB	P32CB
F30CB	P32CG
H31C	S33CA
H31C	S33C
H31CE1	D34CA
H31CE1	D34CB
H31C	D34CB
H31CD2	D34CA
H31CA	S33CB
H31C	S33CB
P32CB	D34CA
P32CG	D34CB
S33N	I35CA
S33CB	I35CA
D34CA	V37CG2
I35CD1	V37CA
I35CD1	V37CG1
I35CG2	V37CB
I35CG2	V37CG1
I35CG2	V37CG2
I35CG1	V37N
D38CA	K41CA
L40CB	G43CA
L40CD1	N42CB
L40CD1	G43CA
L40CG	G43CA
L40CA	N42N
L40CB	N42N
L40CA	G43N
L40CB	G43N

1st nucleus	2nd nucleus
L40CD1	G43N
L40CG	G43N
L40CD1	E44N
L40CD1	E44CG
K41CA	G43CA
K41C	G43CA
N42CB	R45CA
N42CB	E44CA
E44CB	I46CB
E44CA	I46CD1
E44CA	I46CG2
E44CG	I46CD1
E44CB	I46CA
E44CB	I46CD1
E44CB	I46CG2
I46CD1	V49CB
I46CD1	V49CG1
I46CG2	K48CA
I46CG2	V49CB
I46CG2	V49CG1
I46CB	K48CG
I46CB	V49CG1
I46CG1	V49CG1
I46CG1	V49CB
I46CA	K48CG
I46CB	V49CB
I46CA	V49CG1
I46CG2	K48N
I46CG2	K48CB
I46CG2	K48CD
I46CA	K48CA
I46CA	K48CB
I46CB	K48CA
I46CA	K48CD
E47CG	V49CG1
E47CB	V49CG1
V49CA	S52CB
V49CB	S52CA
V49CB	S52CB
V49CG1	S52CA
V49CG1	S52CB
V49CA	S52CA
V49CA	S52N
V49CG1	S52N
V49CG1	D53CA
V49CG1	D53CB
V49CG1	H51CD2
E50CA	S52CA
E50CA	S52CB
E50CB	S52CA
E50CB	S52CB
E50C	S52CB
E50C	S52CA

1st nucleus	2nd nucleus
E50N	S52CB
E50CA	S52N
E50CB	D53CA
S52N	L54CG
S52CB	L54CB
S52CA	L54CB
S52CB	L54CD2
S52CB	L54N
S52CB	L54CD1
S52CB	L54CA
S52CA	L54CD2
S52CB	S55CB
D53CA	S55CB
L54CA	S57CB
L54CD1	F56CB
L54CB	F56CA
L54CD1	F56CG
L54CB	F56CB
S55CB	K58CA
S55CB	K58CD
S55CB	S57CA
S55CB	K58CB
F56CA	W60CG
F56CB	W60CE3
F56CB	W60CG
F56CB	W60CB
F56CA	W60CH2
F56CA	K58CA
F56CG	K58CA
F56CG	W60CA
F56CG	W60CD2
F56CG	W60CH2
F56CA	W60CE3
F56CB	W60CD1
F56CA	W60CE2
F56CB	W60CD2
F56CB	W60CE2
S57CA	W60CA
S57CB	D59CA
S57N	W60CB
S57CB	W60CD1
S57CB	D59CB
D59CA	S61CB
W60CA	F62CB
W60CD1	F62CA
W60CG	F62CA
W60CD1	F62CB
W60CE3	F62CB
W60CA	F62CG
W60CH2	F62CA
W60CE2	F62CA
W60CE2	F62CB
W60CB	F62CB

1st nucleus	2nd nucleus
W60CD1	F62CD2
W60CD2	F62CD2
W60CE3	F62CD2
W60CE2	F62CD2
W60CE2	Y63CA
W60CE2	Y63CB
S61CB	Y63CB
S61CB	L64CA
F62CG	L64CB
F62CB	L64CA
F62CB	L64CD1
F62CG	L65CG
Y63CZ	Y66CA
Y63CZ	Y66CB
L64CA	T68CB
L64CG	Y66CB
L64CA	Y66CB
L64CG	Y66CA
L64CD1	Y66CB
L65CA	T68CB
L65CB	T68CB
L65CD1	T68CG2
L65CD2	T68CG2
L65CD1	T68CB
L65CD2	T68CB
L65CG	T68CA
L65CG	T68CB
L65CB	T68CG2
L65CD2	Y67CA
L65CG	T68CG2
L65CA	T68CG2
L65CD2	T68CA
Y66C	T68CB
T68CG2	F70CB
T68CB	T71C
T68CB	F70CD2
T68CB	F70CE1
E69CA	P72CA
E69CB	T71CB
F70CB	P72CG
F70CA	T73CG2
F70CG	P72CD
F70CA	P72CB
F70CB	P72CA
F70CD2	P72CA
F70CE1	P72CA
F70CE1	P72CB
F70CD2	P72CG
F70CD2	T73CB
P72CA	E74CD
T73CB	K75CD
T73CB	K75CA
E74CB	D76CB

1st nucleus	2nd nucleus
E74CB	D76CA
K75CD	E77CD
K75CE	E77CB
D76CB	Y78CA
D76CA	Y78CD1
E77CB	A79CA
Y78CZ	C80CA
Y78CZ	C80CB
Y78CE1	C80CA
Y78CG	C80CA
Y78CE1	C80CB
A79CA	R81CB
A79CA	R81CD
C80CB	V82CA
C80CB	V82CG1
C80CB	V82CG2
R81CD	N83CA

Supplementary Table 5: Inter-nuclear contacts between atoms within amino acid residues that are far apart in the primary sequence (within >5 residues), observed by MAS-NMR spectroscopy. Contacts coloured RED in Figure 3e.

1st nucleus	2nd nucleus
L23N	V85CG1
L23N	V85CG2
N24CA	V82CG1
N24CB	V82CG1
N24CA	V82CG2
N24CB	V82CG2
N24N	V82CG1
N24N	V82CG2
N24ND2	V82CG1
N24ND2	V82CG2
N24CA	C80CB
N24CB	V85CG1
C25CB	C80CA
C25CB	C80CB
C25CB	V82CA
C25CB	V82CG1
C25N	C80CB
C25N	V82CG1
C25N	V82CG2
C25CB	C80N
C25CA	C80CB
C25CB	V82CG2
C25CB	R81CA
C25CB	Y78CD1
C25CB	A79CA
Y26CB	Y78CZ
V27CA	Y78CB
V27CG1	Y78CE1
V27CA	Y78CG
V27CB	Y78CG
V27CG2	Y78CG
V27CG1	Y78CD2
F30CA	Y78CG
F30CG	Y78CB
F30CG	Y78CE1
F30CG	Y78CG
F30CE1	Y78CA
F30CB	Y78CD1
H31CA	E36CG
H31CE1	E36CA
H31C	D76CG
P32CA	D76CB
P32CB	D76CB
P32CG	D76CA
P32CD	Y78CG
P32CA	D76CA
P32CG	D76CB
P32CB	D76CA
P32CD	T73CB
S33CB	T73CA
S33CB	D76CB
S33CB	T71CB
S33CB	T73CB

1st nucleus	2nd nucleus
I35CD1	L64CA
I35CD1	L64CD1
I35CD1	L64CG
I35CD1	L65CB
I35CD1	F70CB
I35CG2	L64CA
I35CG2	L64CB
I35CG2	L64CD1
I35CG2	L64CG
I35CG2	L65CD2
I35CG2	F70CB
I35CD1	L65CD1
I35CG2	L65CA
I35CA	L64CD1
I35CG1	L65CD1
I35CA	L65CG
I35CG1	L64CD1
I35CG2	F70CD2
V37CG2	F62CG
V37CG1	L64CD2
V37CG2	L64CD2
V37CB	L64CD2
V37CG1	L64CD1
V37CG2	L64CG
V37CA	L64CD1
V37CG1	L64CA
V37CG2	L64CA
V37CG1	F62CD2
V37CB	F62CB
V37CB	L64CA
V37CG1	W60CD1
V37CG1	W60CE2
V37CG2	F62CB
V37CG2	F62CD1
V37CG2	F62CD2
L39CD2	W60CB
L39CB	W60CG
L39CA	W60CD2
L39CB	W60CD2
L39CG	W60CD2
L39CB	W60CB
L39CD1	W60CD1
L39CD1	W60CE2
L39CD1	W60CE3
L40CD1	F56CG
L40CA	F56CE1
L40CG	F56CE1
L40CD1	F56CB
R45CA	L54CD2
I46CA	L54CB
I46CB	L54CB
I46CD1	L54CB
I46CD1	L54CD2

1st nucleus	2nd nucleus
I46CD1	L54CG
I46CG1	S52CB
I46CG1	L54CB
I46CG2	L54CB
I46CG2	L54CD2
I46CG2	L54CG
I46CG1	L54CG
I46CA	L54CG
I46CB	L54CG
I46CA	L54CD1
I46N	L54CG
I46CG1	L54CA
I46CG2	S52CB
I46CA	L54CA
I46CG1	L54CD2
V49CG1	L54CB
V49CG1	L54CD2
V49CG1	L54CG
V49CB	L54CD2
V49CA	L54CD1
V49CA	L54CG
V49CB	L54CA
V49CB	L54CB
V49CB	L54CD1
V49CB	L54CG
V49CG1	L54CA
V49CG1	L54CD1
V49CA	L54CA
V49CA	L54CD2
V49N	L54CB
Y63CB	T68CB
L65CD2	F70CB
L65CB	F70CG
L65CD1	F70CB
L65CA	F70CD2
L65CD1	F70CD2
L65CD2	F70CD2
L65CG	F70CE1

Supplementary Table 6: Analysis of energetic contributions of π-stacking to the β_2m fibril structure

Bond	Face centres distance (Å)	Subunit Contribution (kcal/mol)*	Fibril level contribution (kcal/mol/µm)*
A.Y63 – A.Y66	4.8	-0.9	-1863
A.Y66 – A.Y67	4.8	-0.9	-1863
A.Y67 – B.Y67	7.4	-0.06	-124
B.Y66 – B.Y67	4.8	-0.9	-1863
B.Y63 – B.Y66	4.8	-0.9	-1863

* Estimated based on ring centre distance and data from[6]. Each protofilament within the two-protofilament fibrils is named A or B.

Supplementary Table 7 – Comparison of intrasubunit and interprotofilament interactions in selected amyloid fibril structures in the pdb.

Protein	Tau	Tau	Tau	α-syn	α-syn	α-syn	β_2m	A$\beta_{1\text{-}42}$	A$\beta_{1\text{-}42}$	A$\beta_{1\text{-}42}$	A$\beta_{1\text{-}40}$	A$\beta_{1\text{-}40}$	A$\beta_{1\text{-}40}$
PDB	5o3l	503t	6gx5	6flt	6a6b	2n0a	Xxxx	5kk3	2nao	5oqv	2lmo	2m4j	2mvx
Method	EM[5]	EM[5]	EM[7]	EM[8]	EM[9]	NMR[3]	EM[a]	NMR[10]	NMR[11]	EM[12]	NMR[13]	NMR[14]	NMR[15]
Intrasubunit interactions													
Hydrophobic packing	X	X	X	X	X	X	X	X	X	X		X	X
Steric zippers	X	X		X	X	X	X	X	X	X		X	
Salt Bridges	X	X	X	X	X	X						X	X
π-stacking			X				X						
β-helix	X	X											
Disulfide							X						
Disordered termini	X	X	X	X	X	X	X						
Interprotofilament interactions													
In register		X					X					X	X
Screw axis	X			X	X			X	X	X	X		
Steric zipper				X	X					X			
Backbone H-bonding	X		?										
Terminus salt bridge										X			
Disordered termini	X	X		X	X	n/a[b]	X						
Side chain H-bonding				X	X		X						
π-stacking							X						
Terminus coordination		X											
Hydrophobic packing			X					X	X		X	X	X

[a] This study

[b] Single protofilament structure

Supplementary Table 8: Numbers of multiple ring face to face π-stacking interactions identified in the pdb

**rings involved**	**Count in pdb (of 136,162)**
3	4937
4	521
5	49
6	7
7	3

Supplementary Table 9: Face to face π-stacking interactions involving 5 or more rings found in the pdb

PDB	Type	Description
2f9c	β-helix	YDCK from *Salmonella cholerae*.
2o0w	β-helix	Pectate lyase
2o0v	β-helix	Pectate lyase
2xtw	β-helix	QnrB1
2xty	β-helix	QnrB2
2nzm	β-helix	*Bacillus subtilis* pectate lyase
5ols	β-helix	Rhamnogalacturonan lyase
4fs7	β-helix	leucine-rich repeat protein from *Bacteroides ovatus*
2bsp	β-helix	*Bacillus subtilis* pectate lyase
2pig	β-helix	ydcK from *Salmonella cholerae*
1idj	β-helix	Pectin lyase A
1bn8	β-helix	*Bacillus subtilis* pectate lyase
3krg	β-helix	pectate lyase
2o1d	β-helix	Pectate lyase bound to trisaccharide
2xtx	β-helix	QnrB1
4h09	β-helix	leucine-rich repeat protein from *Eubacterium ventriosum*
1vbl	β-helix	pectate lyase PL 47
3pr7	β-helix	*Moraxella catarrhalis* adhesin UspA1
1dbo	β-helix	chondroitinase B
1dbg	β-helix	chondroitinase B
4gt6	β-helix	leucine rich cell surface protein
1ofl	β-helix	chondroitinase B
1ofm	β-helix	chondroitinase B
5olq	β-helix	rhamnogalacturonan lyase
5olr	β-helix	rhamnogalacturonan lyase
2xt2	β-helix	AlbG
2lmn	fibril z-axis	Aβ1-40
5o3t	fibril z-axis	Straight filament in Alzheimer's disease brain
5o3l	fibril z-axis	Paired helical filament in Alzheimer's disease brain
2mvx	fibril z-axis	Aβ1-420 fibrils: the Osaka mutation
2beg	fibril z-axis	Aβ1-42
5oqv	fibril z-axis	Aβ1-42
5o3o	fibril z-axis	Tau EM
2mxu	fibril z-axis	Aβ1-42
5kk3	fibril z-axis	Aβ1-42
2m5n	fibril z-axis	transthyretin fibrils
5w3n	fibril z-axis	FUS low sequence complexity domain protein fibrils
2mpz	fibril z-axis	Aβ D23N
2n0a	fibril z-axis	α-synuclein
1il5	Lectin-like	ricin A chain bound with inhibitor
1v6n	Lectin-like	Peanut lectin with 9mer peptide
1ioa	Lectin-like	Arcelin-5, a lectin-like defense protein from *Phaseolus vulgaris*
1j1m	Lectin-like	Ricin A-Chain (recombinant) at 100K
5kxc	Lectin-like	*Wisteria floribunda* lectin

1qdo	Lectin-like	Man(α1-3)man(α1-O)methyl concanavilin A complex
1cr7	Lectin-like	Peanut lectin-lactose complex monoclinic form
5clw	Lectin-like	human glycogen branching enzyme in complex with maltoheptaose
1pv6	Lectin-like	lactose permease
1n47	Lectin-like	solectin B4 from *Vicia villosa* in complex with the Tn antigen
5sv3	Lectin-like	RTA1-33/44-198 (RVEC)
4lgp	Lectin-like	ricin A chain bound to camelid nanobody
1br5	Lectin-like	ricin A chain (recombinant) complex with neopterin
3nrb	unique	formyltetrahydrofolate deformylase

Supplementary Table 10: NMR acquisition parameters for β_2m fibrils

3D NCACX, 3D NCOCX, and 3D CONCA spectra (Cambridge Instruments 750 MHz, operating under RNMR[16-20];courtesy of Dr. David Ruben). DARR[21] mixing was used for the 3D NCACX (τ_{mix} = 60 ms) and 3D NCOCX (τ_{mix} = 80 ms). Additionally, 3D NNC$^{\alpha}$ experiments were acquired (Bruker 800 & 900 AVANCE III, with a 3.2 mm triple channel HCN Bruker probe[22]). Spectra were recorded at $\omega_r/2\pi$ = 20 kHz and regulated to ±10 Hz using a Bruker MAS II controller. The 15 ms^{15}N-^{15}N PAR mixing used radio frequency (RF) fields of $\omega_{1H}/2\pi$ =55.4 kHz and $\omega_{15N}/2\pi$ = 32.2 kHz. Spectra at $\omega_{0H}/2\pi$ = 750 MHz were processed with NMRPipe[23], while those at $\omega_{0H}/2\pi$ = 800/900 MHz were processed using TopSpin 3.2. All spectra were analysed in Sparky[24]. ^{13}C and ^{15}N chemical shifts were referenced using the published shifts of adamantine relative to DSS for ^{13}C referencing and the IUPAC relative frequency ratios between DSS (^{13}C) and liquid ammonia (^{15}N)[25]. All experiments were conducted at 268 K

Experiment	Labelling	T [K]	B_0 [T]	ns	ω_r [kHz]	d1 [s]	τ_{mix} [ms]	t_1	sw_1 [Hz]	o_1 [Hz]	t_2	sw_2 [Hz]	o_2 [Hz]	t_3	sw_3 [Hz]	o_3 [Hz]
3D [^{15}N, ^{13}C]-NCACX	[u-^{13}C, u-^{15}N]	268	17.6	16	12.5	2.5	80	3072	100000.00	7524.08	96	4166.70	8338.11	176	12500.00	7524.08
3D [^{15}N, ^{13}C]-NCOCX	[u-^{13}C, u-^{15}N]	268	17.6	16	12.5	2.5	60	2048	100000.00	30660.95	128	4166.70	8338.22	176	6250.00	30660.95
3D [^{15}N, ^{13}C]-CONCA	[u-^{13}C, u-^{15}N]	268	17.6	16	12.5	2.5	-	3072	83333.30	30848.40	128	6250.00	8717.12	128	4166.70	30848.40
3D [^{15}N, ^{13}C]-NNC□	[u-^{13}C, u-^{15}N]	268	18.8	64	20.0	3.0	15	1398	43859.65	12059.50	48	2916.19	9516.96	48	2916.19	9516.96
2D [^{13}C, ^{13}C]-RFDR	[u-^{13}C, u-^{15}N]	268	21.1	32	20.0	2.6	1.6	3072	83333.34	10638.97	1152	50000.00	10638.97	-	-	-
2D [^{13}C, ^{13}C]-PDSD	[u-^{13}C, u-^{15}N]	268	21.1	40	16.0	2.3	600	2400	55555.56	20370.41	1200	55558.22	20370.41	-	-	-
2D [^{13}C, ^{13}C]-PAR	[u-^{13}C, u-^{15}N]	268	21.1	96	20.0	2.4	15	3072	83333.34	11248.99	1536	50000.06	11248.99	-	-	-
2D [^{13}C, ^{15}N]-PAIN	[u-^{13}C, u-^{15}N]	268	21.1	1120	16.0	2.6	12	2560	83333.34	12467.82	192	10000.00	10948.32	-	-	-
2D [^{13}C, ^{13}C]-RFDR	[1,6-^{13}C$_2$-glucose, u-^{15}N]	268	18.8	64	20.0	2.5	1.6	3072	71428.57	20099.17	1024	40000.00	20099.17	-	-	-
2D [^{13}C, ^{13}C]-PDSD	[1,6-^{13}C$_2$-glucose, u-^{15}N]	268	18.8	176	20.0	1.0	1000	2048	62500.00	19094.21	512	40000.00	19094.21	-	-	-
2D [^{13}C, ^{13}C]-PAR	[1,6-^{13}C$_2$-glucose, u-^{15}N]	268	21.1	144	20.0	2.4	15	3072	83333.34	9528.82	1228	49999.68	9528.82	-	-	-
2D [^{13}C, ^{15}N]-PAIN	[1,6-^{13}C$_2$-glucose, u-^{15}N]	268	21.1	1056	20.0	2.6	16	3072	83333.34	9053.00	196	10000.00	11447.00	-	-	-
2D [^{13}C, ^{13}C]-RFDR	[1,3-^{13}C$_2$-glycerol, u-^{15}N]	268	17.6	40	18.2	2.7	1.76	4096	83333.30	18000.00	1080	35714.3	18000.00	-	-	-
2D [^{13}C, ^{13}C]-PDSD	[1,3-^{13}C$_2$-glycerol, u-^{15}N]	268	17.6	64	12.5	2.6	500	4096	83333.30	16000.00	1024	44642.9	16000.00	-	-	-
2D [^{13}C, ^{13}C]-PAR	[1,3-^{13}C$_2$-glycerol, u-^{15}N]	268	21.1	96	20.0	2.4	15	3072	83333.34	9528.82	1228	49999.68	9528.82	-	-	-
2D [^{13}C, ^{15}N]-PAIN	[1,3-^{13}C$_2$-glycerol, u-^{15}N]	268	21.1	960	20.0	2.6	12	3072	83333.34	9053.00	196	10000.00	11493.85	-	-	-
2D [^{13}C, ^{13}C]-PDSD	[2-^{13}C-glycerol, u-^{15}N]	268	17.6	160	16.7	2.4	700	4096	83333.30	18000.00	880	35211.30	18000.00	-	-	-
2D [^{13}C, ^{13}C]-PAR	[2-^{13}C-glycerol, u-^{15}N]	268	21.1	240	20.0	2.4	18	3072	83333.34	11248.99	1536	50000.06	11248.99	-	-	-
2D [^{13}C, ^{15}N]-PAIN	[2-^{13}C-glycerol, u-^{15}N]	268	21.1	896	16.0	2.6	12	3072	83333.34	9053.00	196	10000.00	11447.00	-	-	-
2D [^{13}C, ^{13}C]-RFDR	[^{13}C-VYL, u-^{15}N]	268	21.1	32	16.0	2.5	2	3072	83333.34	9053.00	340	14716.70	9053.00	-	-	-
2D [^{13}C, ^{13}C]-PDSD	[^{13}C-VYL, u-^{15}N]	268	17.6	240	16.0	2.5	200	4096	83333.30	18000.00	800	33783.80	18000.00	-	-	-
2D [^{13}C, ^{13}C]-PAR	[^{13}C-VYL, u-^{15}N]	268	21.1	96	16.0	2.5	15	3072	83333.34	9053.00	340	14716.70	9053.00	-	-	-
2D [^{13}C, ^{15}N]-PAIN	[^{13}C-VYL, u-^{15}N]	268	21.1	992	16.0	2.5	8	3072	83333.34	9053.00	196	10000.00	11448.24	-	-	-

Supplementary Table 11: NMR processing parameters β_2m fibrils

The contact information (Supplementary Tables 2-5) were extracted from a total of 19 2D ^{13}C-^{13}C and ^{13}C-^{15}N correlations. We recorded ^{13}C-^{13}C-PDSD[26], ^{13}C-^{13}C-PAR[27-29], ^{13}C-^{13}C-RFDR[30,31], and ^{13}C-^{15}N-PAIN[32] spectra on almost all isotopically labelled samples.

Experiment	Labelling	SI$_1$	WDW$_1$	LB$_1$ [Hz]	GB$_1$	SSB$_1$	SI$_2$	WDW$_2$	LB$_2$ [Hz]	GB$_2$	SSB$_1$	SI$_3$	WDW$_3$	LB$_3$ [Hz]	GB$_3$	SSB$_3$
3D [^{15}N, ^{13}C]-NCACX	[u-^{13}C, u-^{15}N]	8192	GM	50	-	-	512	GM	50	-	-	512	GM	50	-	-
3D [^{15}N, ^{13}C]-NCOCX	[u-^{13}C, u-^{15}N]	8192	GM	50	-	-	512	GM	50	-	-	512	GM	50	-	-
3D [^{15}N, ^{13}C]-CONCA	[u-^{13}C, u-^{15}N]	8192	GM	60	-	-	512	GM	55	-	-	256	GM	60	-	-
3D [^{15}N, ^{13}C]-NNC□	[u-^{13}C, u-^{15}N]	8192	GM	20	0.03	-	256	EM	50	-	-	256	GM	20	0.03	-
2D [^{13}C, ^{13}C]-RFDR	[u-^{13}C, u-^{15}N]	8192	GM	80	-	-	4096	GM	80	-	-	-	-	-	-	-
2D [^{13}C, ^{13}C]-PDSD	[u-^{13}C, u-^{15}N]	8192	GM	80	0.1	-	2048	GM	80	0.1	-	-	-	-	-	-
2D [^{13}C, ^{13}C]-PAR	[u-^{13}C, u-^{15}N]	8192	GM	50	0.1	-	2048	GM	50	0.1	-	-	-	-	-	-
2D [^{13}C, ^{15}N]-PAIN	[u-^{13}C, u-^{15}N]	8192	GM	20	0.03	-	1024	GM	20	0.03	-	-	-	-	-	-
2D [^{13}C, ^{13}C]-RFDR	[1,6-$^{13}C_2$-glucose, u-^{15}N]	8192	GM	50	0.1	-	2048	GM	50	0.1	-	-	-	-	-	-
2D [^{13}C, ^{13}C]-PDSD	[1,6-$^{13}C_2$-glucose, u-^{15}N]	8192	GM	20	0.05	-	2048	GM	20	0.05	-	-	-	-	-	-
2D [^{13}C, ^{13}C]-PAR	[1,6-$^{13}C_2$-glucose, u-^{15}N]	8192	QSINE	-	-	3.5	2048	QSINE	-	-	3.5	-	-	-	-	-
2D [^{13}C, ^{15}N]-PAIN	[1,6-$^{13}C_2$-glucose, u-^{15}N]	8192	GM	80	-	-	512	GM	80	-	-	-	-	-	-	-
2D [^{13}C, ^{13}C]-RFDR	[1,3-$^{13}C_2$-glycerol, u-^{15}N]	8192	GM	80	-	-	2048	GM	80	-	-	-	-	-	-	-
2D [^{13}C, ^{13}C]-PDSD	[1,3-$^{13}C_2$-glycerol, u-^{15}N]	8192	GM	80	-	-	2048	GM	80	-	-	-	-	-	-	-
2D [^{13}C, ^{13}C]-PAR	[1,3-$^{13}C_2$-glycerol, u-^{15}N]	8192	GM	80	-	-	4096	GM	80	-	-	-	-	-	-	-
2D [^{13}C, ^{15}N]-PAIN	[1,3-$^{13}C_2$-glycerol, u-^{15}N]	8192	GM	80	-	-	512	GM	80	-	-	-	-	-	-	-
2D [^{13}C, ^{13}C]-PDSD	[2-^{13}C-glycerol, u-^{15}N]	8192	GM	80	-	-	2048	GM	80	-	-	-	-	-	-	-
2D [^{13}C, ^{13}C]-PAR	[2-^{13}C-glycerol, u-^{15}N]	8192	GM	80	-	-	4096	GM	80	-	-	-	-	-	-	-
2D [^{13}C, ^{15}N]-PAIN	[2-^{13}C-glycerol, u-^{15}N]	8192	GM	20	0.07	-	1024	GM	20	0.07	-	-	-	-	-	-
2D [^{13}C, ^{13}C]-RFDR	[^{13}C-VYL, u-^{15}N]	8192	GM	80	-	-	1024	GM	80	-	-	-	-	-	-	-
2D [^{13}C, ^{13}C]-PDSD	[^{13}C-VYL, u-^{15}N]	8192	GM	50	-	-	2048	GM	50	-	-	-	-	-	-	-
2D [^{13}C, ^{13}C]-PAR	[^{13}C-VYL, u-^{15}N]	8192	GM	80	-	-	1024	GM	80	-		-	-	-	-	-
2D [^{13}C, ^{15}N]-PAIN	[^{13}C-VYL, u-^{15}N]	8192	GM	80	-	-	512	GM	80	-		-	-	-	-	-

Supplementary Table 12: Refinement statistics for the final β_2m two protofilament fibril model

Data collection	Dataset 1	Dataset 2
Micrographs	2019	3530
Defocus range (µM)	-1.75 to -3.75 µm	-1.75 to -3.75 µm
Voltage (kV)	300	300
Exposure time (s)	10	10
Number of frames	40	40
Dose/ frame (e^-/Å^2)	1.05	0.89
Total dose(e^-/Å^2)	42.3	35.8
Pixel size (Å)	1.06	1.06

Refinement statistics	
Box size (px)	300
Box overlap (px)	270
Individual fibrils	622
Particles	11035
Subunit rise (Å)	4.83
Subunit Twist (˚)	-0.608
Resolution (Å)	3.9
B-factor (Å^2)	-160.013

Model statistics	
β_2m Subunits	8
Clash score	29.5
Poor rotomers	0%
Favored rotomers	90.98%
Ramachandran outliers	0%
Ramachandran favored	88.71%
Molprobity score	2.54
Bad bonds	0%
Bad angles	0%
Cis prolines	0 / 2
CaBLAM outliers	4.99%
CA geometry outliers	0%

References

1. Lawrence, M.C. & Colman, P.M. Shape complementarity at protein/protein interfaces. *J. Mol. Biol.* **234**, 946-950 (1993).
2. Michel, G. et al. The structure of chondroitin B lyase complexed with glycosaminoglycan oligosaccharides unravels a calcium-dependent catalytic machinery. *J. Biol. Chem.* **279**, 32882-32896 (2004).
3. Tuttle, M.D. et al. Solid-state NMR structure of a pathogenic fibril of full-length human alpha-synuclein. *Nat. Struct. Mol. Biol.* **23**, 409-415 (2016).
4. Ravishankar, R., Thomas, C., Suguna, K., Surolia, A. & Vijayan, M. Crystal structures of the peanut lectin-lactose complex at acidic pH: retention of unusual quaternary structure, empty and carbohydrate bound combining sites, molecular mimicry and crystal packing directed by interactions at the combining site. *Proteins* **43**, 260-270 (2001).
5. Fitzpatrick, A.W.P. et al. Cryo-EM structures of tau filaments from Alzheimer's disease. *Nature* **547**, 185-190 (2017).
6. McGaughey, G., Gagné, M. & Rappé, A. π-stacking interactions: alive and cell in proteins. *J. Biol. Chem.* **273**, 15458-15463 (1998).
7. Falcon, B. *et al.* Structures of filaments from Pick's disease reveal a novel tau protein fold. *Nature* **561,** 137–140 (2018).
8. Guerrero-Ferreira, R. et al. Cryo-EM structure of alpha-synuclein fibrils. *Elife* **7**, e36402 (2018).
9. Li, Y. et al. Amyloid fibril structure of alpha-synuclein determined by cryo-electron microscopy. *Cell Res* (2018).
10. Colvin, M.T. et al. Atomic resolution structure of monomorphic Aβ42 amyloid fibrils. *J. Am. Chem. Soc.* **138**, 9663-9674 (2016).
11. Walti, M.A. et al. Atomic-resolution structure of a disease-relevant Aβ(1-42) amyloid fibril. *Proc. Natl. Acad. Sci. USA* **113**, E4976-E4984 (2016).
12. Gremer, L. et al. Fibril structure of amyloid-β(1-42) by cryo-electron microscopy. *Science* **358**, 116-119 (2017).
13. Paravastu, A.K., Leapman, R.D., Yaua, W.-M. & Tycko, R. Molecular structural basis for polymorphism in Alzheimer’s β-amyloid fibrils. *Proc. Natl. Acad. Sci. USA* **105**, 18349-18354 (2008).
14. Lu, J.X. et al. Molecular structure of β-amyloid fibrils in Alzheimer's disease brain tissue. *Cell* **154**, 1257-1268 (2013).
15. Schutz, A.K. et al. Atomic-resolution three-dimensional structure of amyloid beta fibrils bearing the Osaka mutation. *Angew. Chem. Int. Ed. Engl.* **54**, 331-335 (2015).
16. Igumenova, T.I., Wand, A.J. & McDermott, A.E. Assignment of the backbone resonances for microcrystalline ubiquitin. *J. Am. Chem. Soc.* **126**, 5323-5331 (2004).
17. Marulanda, D. et al. Magic angle spinning solid-state NMR spectroscopy for structural studies of protein interfaces. resonance assignments of differentially enriched Escherichia coli thioredoxin reassembled by fragment complementation. *J. Am. Chem. Soc.* **126**, 16608-16620 (2004).
18. Pauli, J., Baldus, M., van Rossum, B., de Groot, H. & Oschkinat, H. Backbone and side-chain ^{13}C and ^{15}N signal assignments of the alpha-spectrin SH3 domain by magic angle spinning solid-state NMR at 17.6 Tesla. *Chembiochem* **2**, 272-281 (2001).
19. Shi, L. et al. Three-dimensional solid-state NMR study of a seven-helical integral membrane proton pump-structural insights. *J. Mol. Biol.* **386**, 1078-1093 (2009).
20. Sperling, L.J., Berthold, D.A., Sasser, T.L., Jeisy-Scott, V. & Rienstra, C.M. Assignment strategies for large proteins by magic-angle spinning NMR: the 21-kDa disulfide-bond-forming enzyme DsbA. *J. Mol. Biol.* **399**, 268-282 (2010).
21. Takegoshi, K., Nakamura, S. & Terao, T. ^{13}C-^{1}H dipolar-assisted rotational resonance in magic-angle spinning NMR. *Chem. Phys. Lett.* **344**, 631-637 (2001).

22. Donovan, K.J., Silvers, R., Linse, S. & Griffin, R.G. 3D MAS NMR Experiment utilizing through-space (15)N-(15)N correlations. *J. Am. Chem. Soc.* **139**, 6518-6521 (2017).
23. Delaglio, F. et al. NMRpipe - A multidimensional spectral processing system Based on Unix Pipes. *J. Biomol. NMR* **6**, 277-293 (1995).
24. Goddard, T.D. & Kneller, D.G. Sparky 3. (University of California, San Francisco).
25. Morcombe, C.R. & Zilm, K.W. Chemical shift referencing in MAS solid state NMR. *J. Magn. Reson.* **162**, 479-486 (2003).
26. Bloembergen, N. On the interaction of nuclear spins in a crystalline lattice. *Physicia* **XV**, 386-426 (1949).
27. De Paëpe, G., Lewandowski, J.R., Loquet, A., Bockmann, A. & Griffin, R.G. Proton assisted recoupling and protein structure determination. *J. Chem. Phys.* **129**, 245101 (2008).
28. Lewandowski, J.R. et al. Proton assisted recoupling at high spinning frequencies. *J. Phys. Chem. B* **113**, 9062-9069 (2009).
29. Donovan, K.J., Jain, S.K., Silvers, R., Linse, S. & Griffin, R.G. Proton-assisted recoupling (PAR) in peptides and proteins. *J. Phys. Chem. B* **121**, 10804-10817 (2017).
30. Bennett, A.E., Griffin, R.G., Ok, J.H. & Vega, S. Chemical shift correlation spectroscopy in rotating solids: Radio frequency-driven dipolar recoupling and longitudinal exchange. *J. Chem. Phys.* **96**, 8624-8627 (1992).
31. Bennett, A.E. et al. Homonuclear radio frequency-driven recoupling in rotating solids. *J. Chem. Phys.* **108**, 9463-9479 (1998)
32. Lewandowski, J.R., De Paëpe, G. & Griffin, R.G. Proton assisted insensitive nuclei cross polarization. *J. Am. Chem. Soc.* **129**, 728-729 (2007).
