# Supplementary material for: The structure of a β2-microglobulin fibril suggests a molecular basis for its amyloid polymorphism
Source: Nat Commun. 2018 Oct 30;9:4517. doi: 10.1038/s41467-018-06761-6 (PMC6207761; doi:10.1038/s41467-018-06761-6)
Supplement: Supplementary file 2 — Description of Additional Supplementary Files [file 41467_2018_6761_MOESM2_ESM.pdf]

Description of Additional Supplementary Files

File Name: Supplementary Movie 1
Description: The 3.9Å structure of a two protofilament β2m amyloid fibril structure. The two protofilaments are coloured pink and blue, and the fibril rotates around it's long axis.

File Name: Supplementary Movie 2
Description: The atomic model of a two protofilament β2m fibril. The starting view represents a cross section through a layer of the EM reconstruction, perpendicular to the long fibril axis. Each β2m subunit is L-shaped, stacked in register, perpendicular to the fibril long (C2) axis, and related by a 180° rotation about the C2 axis. The polypeptide is shown in cartoon representation, coloured from the Nterminus (i.e. M0 would be deep blue) to the C-terminus (M99 would be deep red). Side-chains are coloured in a CPK scheme. In sequence, the following features are highlighted: A steric zipper in the foot; a β-turn in the toe; the disulfide-containing region; and the interface between protofilaments showing π-stacking.
